# Lipid Systems for the Delivery of Amphotericin B in Antifungal Therapy

**DOI:** 10.3390/pharmaceutics12010029

**Published:** 2020-01-01

**Authors:** Célia Faustino, Lídia Pinheiro

**Affiliations:** Research Institute for Medicines (iMed.ULisboa), Faculty of Pharmacy, Universidade de Lisboa, Av. Prof. Gama Pinto, 1649-003 Lisboa, Portugal; cfaustino@ff.ulisboa.pt

**Keywords:** amphotericin B, fungal diseases, drug delivery, lipid systems, nanoparticles, infection

## Abstract

Amphotericin B (AmB), a broad-spectrum polyene antibiotic in the clinic for more than fifty years, remains the gold standard in the treatment of life-threatening invasive fungal infections and visceral leishmaniasis. Due to its poor water solubility and membrane permeability, AmB is conventionally formulated with deoxycholate as a micellar suspension for intravenous administration, but severe infusion-related side effects and nephrotoxicity hamper its therapeutic potential. Lipid-based formulations, such as liposomal AmB, have been developed which significantly reduce the toxic side effects of the drug. However, their high cost and the need for parenteral administration limit their widespread use. Therefore, delivery systems that can retain or even enhance antimicrobial efficacy while simultaneously reducing AmB adverse events are an active area of research. Among those, lipid systems have been extensively investigated due to the high affinity of AmB for binding lipids. The development of a safe and cost-effective oral formulation able to improve drug accessibility would be a major breakthrough, and several lipid systems for the oral delivery of AmB are currently under development. This review summarizes recent advances in lipid-based systems for targeted delivery of AmB focusing on non-parenteral nanoparticulate formulations mainly investigated over the last five years and highlighting those that are currently in clinical trials.

## 1. Introduction

Fungal diseases affect over a billion people worldwide, being responsible for more than 1.5 million deaths each year [1]. Severity may range from asymptomatic and mild cutaneous and mucosal infections to chronic diseases and life-threatening systemic infections [1,2]. The highest burdens are associated with recurrent vulvovaginal candidiasis that affects approximately 138 million women annually and allergic fungal diseases (“fungal asthma”) with a prevalence estimate of more than 10 million people per year [3]. Morbidity and mortality due to fungal diseases are higher in low- and middle-income countries due to lack of, or restricted access to, rapid and reliable fungal diagnostic tools, late or inaccurate diagnosis, and limited availability of life-saving antifungal drugs [4].

Fungal diseases can also affect plants and animals and are often caused by yeasts or molds commonly encountered in the environment. *Candida albicans*, *Trichophyton rubrum,* and *Aspergillus fumigatus* are the main pathogenic agents responsible for most mucosal, skin, and allergic human fungal diseases, respectively [5]. Nevertheless, fungal pathogens remain mostly neglected by public health authorities and research funding bodies, despite their strong impact on human health and crop production that results in high social and economic burdens [1,6].

Many fungal diseases are opportunistic infections that can be fatal to immunocompromised patients, such as people with human immunodeficiency virus (HIV)/acquired immunodeficiency syndrome (AIDS), organ and stem cell transplant recipients, cancer patients, and those on long-term corticosteroid therapy [1,2,7]. Cryptococcal meningitis, *Pneumocystis* pneumonia and disseminated histoplasmosis are major AIDS-associated fungal diseases with a high mortality rate if not diagnosed or treated while chronic pulmonary aspergillosis is a usual complication following tuberculosis and other lung diseases [8].

Hospitalized patients are also at a higher risk of developing a fungal infection. Life-threatening invasive candidiasis and invasive aspergillosis are among the most common healthcare-associated infections (HAIs), requiring longer hospitalization stay and often expensive antifungal drugs, thus contributing to increased healthcare costs [7,9,10]. Candida bloodstream infections, which have mortality rates around 50%, are HAIs frequently related with the use of central venous catheters (CVCs) and treatment often requires catheter removal due to formation of recalcitrant biofilms [10,11,12].

Biofilm formation, an important virulence factor for pathogenic fungi, often contributes to the development of antimicrobial resistance [11,12,13,14]. Fungal biofilms are surface-associated communities of microbial cells protected by an extracellular polysaccharide-rich matrix that inhibits diffusion and cell uptake of antimicrobial agents [11,12,15]. Decreased susceptibility to antifungal drugs results in higher minimum inhibitory concentration (MIC) values for microbial strains grown as biofilms compared to their corresponding planktonic forms [15]. This often hampers therapeutic options and contributes to the emergence and spread of antibiotic resistance [12].

Many cutaneous implantation mycoses, such as sporotrichosis, chromoblastomycosis, mycetoma, and fungal keratitis, are neglected diseases that prevail in tropical or subtropical regions [4,16]. Recently, the World Health Organization (WHO, *Geneva,* Switzerland) included mycetoma and chromoblastomycosis in the list of neglected tropical diseases [17]. Paracoccidioidomycosis is still one of the most prevalent systemic mycosis endemics in Latin America [6,16]. However, climate changes, traveler increase, human migration, and intensive fungicide use in food crops is shifting the epidemiology of these infections [10,18,19].

Antifungal agents included in the current WHO Model List of Essential Medicines are limited to orally available azoles (clotrimazole, fluconazole, itraconazole, and voriconazole), polyene antibiotics (nystatin and amphotericin B), the antimetabolite flucytosine, and the microtubule inhibitor griseofulvin [20]. The azoles are the most widely used antifungal agents in the clinic, also employed for crop protection and livestock treatment. The emergence of azole-resistant *Aspergillus* strains [19,21] and multidrug resistant (MDR) *Candida auris* [9,10,19] represents a serious and global health threat since antifungal vaccines are lacking and the latest clinical antifungal agents introduced in the market were the echinocandin lipopeptides (caspofungin, micafungin, and anidulafungin) in the beginning of the century [22].

Although oral azoles (usually itraconazole or fluconazole) are still the recommended drugs for most mild fungal diseases, intravenous (i.v.) amphotericin B (AmB) remains the drug of choice for invasive fungal infections, MDR fungal pathogens (resistant to both fluconazole and an echinocandin), and visceral leishmaniasis (kala-azar) [4,16,22]. Despite being part of the WHO list of essential medicines since 2013, AmB is still not available in many countries, including some where fungal diseases have high mortality rates [4].

## 2. Amphotericin B Properties and Mode of Action

Amphotericin B, a macrolide polyene antibiotic produced by *Streptomyces nodosus*, has been considered the gold standard drug for the treatment of severe systemic fungal infections since its introduction in the market back in 1958, mainly due to its broad spectrum of activity and low frequency of resistance development [22,23,24,25]. AmB is effective in the treatment of aspergillosis [26], candidiasis [27], blastomycosis [28], paracoccidioidomycosis [29], coccidioidomycosis [28], cryptococcosis [30], histoplasmosis [28], mucormycosis [31], some hyalohyphomycosis [32] and phaeohyphomycosis [33], dermatophytosis [34] and other dermatomycosis [35], sporotrichosis [36], talaromycosis (formerly penicilliosis) [37], and trichosporonosis [38]. The drug is also active against some parasitic diseases, namely leishmaniasis (cutaneous, mucocutaneous, and visceral) [22,39] and primary amebic meningoencephalitis [22,40].

**Figure 1:**
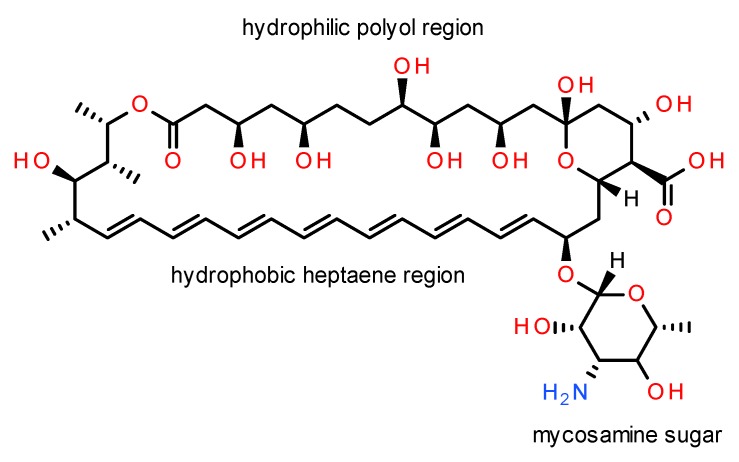
Chemical structure of amphotericin B.

AmB is a macrocyclic lactone with amphiphatic and amphoteric properties due to the presence of hydrophobic polyene and hydrophilic polyol regions, attached to both a carboxylic acid group (p*K*_a_ 5.7), and a basic mycosamine (p*K*_a_ 10) sugar (Figure 1). AmB can be either fungistatic or fungicidal depending on fungal susceptibility, drug concentration, and pH, achieving maximum antifungal activity at pH 6.0–7.5 [25,41,42]. However, the high molecular weight of the drug (*M*_r_ 924.08) and its reduced solubility and permeability contribute to its poor pharmacokinetic profile [25,41,42]. AmB is also unstable in acid media, sensitive to light and temperature [25,42], requiring storage between 2–8 °C.

Due to its amphipathic nature, AmB is able to self-associate in aqueous solution forming water soluble dimers and oligomers that can further associate to form insoluble polyaggregates, which act as a monomer reservoir [25,43,44,45,46]. The nature and proportion of each species in both aqueous and lipid phases is dependent on total drug concentration, temperature, type of formulation, and membrane composition, being correlated with AmB efficacy and toxicity [43,44,45,46,47,48,49,50,51]. Drug morphology (crystalline or amorphous state) and formulation techniques also influence the rate of dissolution and solubility of AmB [52].

The antibiotic targets the cellular membrane, showing higher affinity for ergosterol-containing membranes typical of fungal cells than for cholesterol-containing membranes of mammalian host cells [44,53,54]. AmB oligomers are particularly toxic to eukaryotic cells leading to high antifungal activity but also severe toxic side effects [43,44,45,46,48,49,51,55] while polyaggregated and monomeric forms of the drug retain antifungal activity and show reduced toxicity towards host cells [43,49,51]. This suggests that better selectivity for fungal cells leading to improved therapeutic index may be achieved by carefully controlling the aggregation state of the drug [25,43,45,46], which can be easily determined from AmB ultraviolet (UV) absorption or fluorescence spectra that are sensitive to different aggregation states [46,56]. Self-association of the drug is related to sequestration of the polyene chromophore within a more hydrophobic environment and the polyene vibronic structure of monomeric AmB collapses to a blue-shifted band typical of aggregated structures [25,57].

Despite several decades of clinical use, AmB mechanism of action at the molecular level remains elusive and several models have been proposed based on extensive experimental research and theoretical studies [45,46,47,48,54,55,58,59,60,61,62,63,64,65,66]. AmB has been shown to bind sterol-containing membranes of eukaryotic cells and to insert into the lipid bilayer forming pore-like supramolecular structures that can act as transmembrane ion channels, leading to increased membrane permeability, K^+^ leakage, and disruption of ion transport [44,45,48,53]. Recent studies also suggest that different oligomerization of AmB in lipid bilayers modulated by membrane sterols contribute to the higher toxicity of the drug to fungal cells, since it has been found that ergosterol promotes association of AmB dimers into tetramers responsible for membrane permeabilization while cholesterol hinders AmB aggregation in the lipid matrix [44,46,48,55,62]. Disruption of ergosterol biosynthesis is responsible for resistance to AmB in *Candida lusitaniae* [67] and for cross resistance to azoles and AmB in a clinical isolate of *C. albicans* [68].

However, it was determined that channel formation is not required for fungicidal activity and an alternative mechanism based on direct binding and sequestration of membrane ergosterol has been proposed, suggesting that the pore-inducing ability of AmB could be separated from its cytocidal effects [60]. Recently, it was suggested that AmB can form large extramembranous aggregates that act as fungicidal sterol sponges by extracting ergosterol from lipid bilayers [58]. Sequestration of ergosterol by AmB, either at the membrane surface (surface adsorption model) or in the form of extracellular aggregates (sterol sponge model), destabilizes the lipid phase and disrupts the structural integrity of the lipid bilayer resulting in impaired membrane functionality, which may underlie the resistance-refractory antimicrobial action of AmB [44,48,58,59,60]. In the same context, sequestration of the host membrane cholesterol avoiding macrophage–parasite interaction has been proposed as an alternative mode of action for AmB in visceral leishmaniasis (VL) [69].

On the other hand, imaging of both normal epithelial and colon adenocarcinoma human cells exposed to AmB revealed a detoxifying mechanism based on the formation of AmB-containing exosomes devoid of cholesterol, suggesting that insertion of the drug within the hydrophobic membrane core is sufficient to disturb the membrane structure and lead to cytotoxic effects [61]. The fungicidal activity of AmB has also been attributed to vacuole disintegration resulting from trafficking of the drug to the vacuolar lumen via autophagy [70]. Moreover, oxidative cell damage to the lipid membrane that results from increased mitochondrial production and intracellular accumulation of reactive oxygen species (ROS) induced by AmB leads to impaired cellular functions and also contributes to the fungicidal activity of the drug [59,63,64,65,66]. Better microbial adaptation to oxidative stress has been suggested to contribute to the development of AmB tolerance in some *Aspergillus terreus* strains [71].

AmB also has immunomodulatory properties in mammalian host cells which can enhance the immune system of the host and elicit inflammatory responses that depend on AmB formulation and may involve stimulation of cytokine, chemokine, prostaglandin, ROS, and/or nitric oxide production [72,73]. The immunomodulatory activity of AmB is mediated via Toll-like receptors (TLRs) and the co-receptor CD14 [74,75]. AmB-induced elevated levels of pro-inflammatory mediators, such as tumor necrosis factor (TNF)-α and interleukin (IL)-1β, IL-6, and IL-8, have been associated with several toxic side effects of the drug [72,73,76].

## 3. Commercial Amphotericin B Lipid Formulations

AmB has very low water solubility and membrane permeability, thus poor oral bioavailability, since it was originally formulated as a colloidal suspension for parenteral administration using sodium deoxycholate (a bile salt detergent) as the solubilizing agent [24,77,78]. This conventional formulation of AmB deoxycholate (AmB-DOC, Fungizone^®^) forms a micellar suspension when reconstituted in 5% dextrose solution prior to i.v. administration. Upon dilution in the plasma, it rapidly releases AmB, mostly in the form of toxic oligomeric aggregates [43,77]. The drug mainly accumulates in the liver, spleen, kidneys, and lungs, being slowly excreted unchanged via the urinary and biliary routes [24,25,79].

Despite its efficacy, AmB-DOC has a narrow therapeutic window due to dose-dependent adverse events, particularly severe nephrotoxicity [24,46,77], including renal vasoconstriction and decreased glomerular filtration rate [80,81]. AmB is extensively bound to plasma lipoproteins showing preference for low-density lipoproteins (LDLs) over high-density lipoproteins (HDLs) [79,82]. The uptake of the LDL-AmB complexes through receptor-mediated endocytosis by renal tubular cells (with low expression of HDL receptors) strongly contributes to the drug nephrotoxicity [24,83].

Other common AmB side effects include cardiovascular, hepatic, and hematopoietic disorders as well as acute infusion-related reactions, such as fever, chills, hypotension, nausea, vomiting, headache, tachypnea, arrhythmias, rash, (thrombo)phlebitis, and injection site pain [24,46,77]. Many of these side effects have been associated with the pro-inflammatory response induced by AmB through the stimulation of TLR2 or CD14 co-receptor [74,75]. At concentrations typically found in the human serum, AmB-DOC promotes production of pro-inflammatory cytokines (TNF-α) and chemokines (IL-8) in human monocytic THP-1 and kidney HEK293 cell lines [75]. Furthermore, patients receiving AmB-DOC showed persistently elevated levels of pro-inflammatory cytokines linked with the development of drug-induced kidney damage [76].

Mild heating of Fungizone^®^ (20 min at 70 °C) was found to increase the thermodynamic stability of the formulation and to improve its therapeutic index by producing a super aggregated and less toxic form of AmB while retaining antifungal efficacy [57,84,85,86,87,88,89,90]. Heat-induced superaggregation of AmB was shown to modify its distribution among the serum lipoproteins and to attenuate AmB-stimulated production of TNF-α and other pro-inflammatory mediators in human THP-1 monocytes in vitro [86,91], which may contribute to its reduced cytotoxicity against host cells in vivo in experimental animal mycoses [86,87,89,92]. The increase in particle size, from ca 4 nm thread-like micelles in Fungizone^®^ to ca 300 nm cobweb-like structures in the heat-treated formulation [89], promoted macrophage uptake and improved efficacy against *Leishmania donovani*, both in vitro [93] and in vivo [93,94].

Alternative parenteral formulations employing lipid vehicles for AmB delivery were developed in order to improve drug tolerability and optimize its clinical efficacy [79,95]. Three of such lipid-based formulations reached the market in the 1990s after approval by the United States Food and Drug Administration (FDA, Silver Spring, MD, USA) and the European Medicines Agency (EMA, Amsterdam, the Netherlands) [25,79], remaining commercially available in several countries:

- Amphotericin B lipid complex (ABLC, Abelcet^®^), consisting of microscopic ribbon-like lipid structures;
- Amphotericin B colloidal dispersion (ABCD, Amphotec^®^/Amphocil^®^), in which the drug forms disk-shaped lipid structures with sodium cholesteryl sulfate, a naturally occurring cholesterol metabolite;
- Liposomal Amphotericin B (L-AmB, AmBisome^®^), in which the drug is intercalated within the lipid bilayer of cholesterol-containing liposomes.

AmB lipid formulations exhibit distinct pharmacokinetic profiles (Table 1) and are not interchangeable [95,96,97,98,99,100,101,102,103,104,105,106], having different dosing recommendations.

All commercial lipid formulations demonstrated a safer profile compared to conventional AmB (Fungizone^®^) and similar therapeutic efficacy (although at larger doses) in preclinical and clinical studies [79,107,108,109], but these differences appear to be less marked in the pediatric population [110]. The lipid vehicle allows selective and controlled release of the drug to fungal cells while preventing its interaction with membrane cholesterol of the host cells, thus reducing the drug side effects [25,42,79,80,96,111]. Recent electron microscopy studies performed by Walker et al. demonstrated that the viscoelastic properties of the fungal cell wall allowed traffic of AmBisome^®^ as intact liposome vesicles [112]. At the target site, the higher affinity of AmB for ergosterol over the lipid vehicle [113] and the presence of lipases from fungal or inflammatory host cells (or phagocytic digestion by infected macrophages in leishmaniasis) promoted the release of monomeric AmB from the lipid complex and binding of the drug to the cell membrane of the pathogen [96]. Moreover, AmB lipid formulations are also more efficient at biofilm penetration than conventional AmB-DOC and have shown enhanced antifungal activity against *Candida* spp. biofilms in vitro [15,114,115] and in vivo in animal models of catheter-associated *Candida* biofilm infection [114,116,117]. Pilot studies demonstrating the feasibility of L-AmB lock therapy in combination with systemic antifungal therapy for catheter salvage in patients with CVC-related candidemia have also been reported [118,119].

The reticuloendothelial system (RES) is responsible for the rapid plasma clearance of large colloidal particles that further accumulate in the liver and spleen while smaller particles, such as the small liposomes in the AmBisome^®^ formulation, can escape RES and have prolonged blood circulation half-life (Table 1) [25,42,77,79]. The high transition temperature of the liposome phospholipid components also contributes to the physiological stability of L-AmB [25,79]. Tissue concentration of AmB in autopsy samples of patients treated with AmB lipid formulations for suspected or proven invasive fungal infection showed the highest AmB levels in the liver and spleen followed by kidney, lung, myocardium, and brain [120]. Biodistribution studies in noninfected rabbits showed that high AmB concentrations were achieved in the liver and bone marrow after seven days of treatment with the lipid formulations (L-AmB, ABLC, and ABCD) at 5 mg/kg/day while concentrations of the drug in fat tissue were generally low, supporting the involvement of the mononuclear phagocytic system in this preferential distribution pattern [121]. AmB concentrations in plasma, cerebrospinal fluid (CSF), and brain tissue at 30 min after the last dose were higher for L-AmB but did not result in enhanced CSF penetration compared to AmB-DOC at 1 mg/kg/day [122]. In a rabbit model of hematogenous *C. albicans* meningoencephalitis, treatment with L-AmB (5 mg/kg/day) or AmB-DOC (1 mg/kg/day) resulted in complete eradication of *C. albicans* from brain tissue whereas ABLC and ABCD treatment were only partially effective [122]. Concentration gradients were suggested as the major determinants for AmB delivery to the central nervous system (CNS), with eventual contribution of drug leakage from the delivery vehicle in damaged endothelium due to infection and/or inflammation [122].

Compared to conventional AmB-DOC, the larger particle size of the lipid formulations prevents glomerular filtration, which results in decreased nephrotoxicity [25,42,79]. Furthermore, lipid-based AmB formulations (but not AmB-DOC) promote AmB transfer into serum HDLs by increasing the activity of phospholipid transfer proteins (PLTPs) and inhibiting cholesteryl ester transfer protein (CETP)-mediated transfer from HDLs to LDLs, resulting in reduced uptake by renal cells and thus lower nephrotoxicity when compared to Fungizone^®^ [83,88,123]. It has been suggested that plasma lipid levels may influence the distribution of AmB from lipid-based formulations into different serum lipoprotein fractions [123].

Differential expression of inflammatory mediators induced by AmB in conventional and lipid formulations are also responsible for attenuation of the drug adverse events in the latter. In vitro studies in rat alveolar macrophage cells exposed to AmB lipid formulations showed significantly decreased production of nitric oxide compared to lipopolysaccharide (LPS) [124]. L-AmB was found to alter the immune response in an in vitro sepsis model by modulating the pro-inflammatory cytokine gene and protein expression levels and phagocytic activity of LPS-stimulated human monocytes [125]. However, in human monocytes, ABCD (and AmB-DOC) upregulated the production of pro-inflammatory mediators (contrary to ABLC and L-AmB) resulting in frequent infusion-related toxic side effects [126] that led to premature termination of a randomized clinical trial of ABCD (4 mg/kg/day) for antifungal prophylaxis in neutropenia patients with hematological malignancies [127]. Pre-medication with corticosteroids (but not with paracetamol or antihistamines) is associated with a decreased incidence of infusion-related reactions in patients receiving ABCD (Amphotec^®^) infusions [128].

Among the lipid formulations, L-AmB (AmBisome^®^) is associated with fewer and less frequent infusion-related reactions and nephrotoxicity adverse events [80,107,108,129]. L-AmB was shown to activate murine neutrophils against *A. fumigatus* by diverting Toll-like receptor signaling from TLR-2 to TLR-4, leading to preferential release of anti-inflammatory cytokine IL-10 over pro-inflammatory TNF-α, the latter associated with TLR2 binding [74]. This liposome-mediated effect has been attributed to efficient phagocytic uptake of the small and negatively charged liposomes of L-AmB, and the liposomes alone were able to change the cellular response from pro- to anti-inflammatory [74].

However, due to the high cost of the lipid formulations, in resource-limited settings that rely only on the conventional AmB formulation for the treatment of systemic fungal infections, extemporaneous fat emulsions have been alternatively prepared by mixing AmB-DOC with Intralipid^®^ 20%, a low-cost commercial water-in-oil (O/W) emulsion for parenteral nutrition containing 20% (*w*/*v*) soybean oil [130,131]. AmB-DOC in Intralipid^®^ (AmB-IL) has shown antifungal efficacy in vitro [132] and in vivo in experimental animal models of systemic candidiasis [133,134] and aspergillosis [135], similar to that of the conventional formulation in dextrose but decreased nephrotoxicity and infusion-related side effects. Improved tolerance may be due to decreased expression of pro-inflammatory cytokines TNF-α and IL-1β found in mice with invasive fungal infections treated with AmB-IL when compared with dextrose infusions of AmB-DOC [134]. In preclinical and clinical studies, the AmB-IL admixture showed a different pharmacokinetic profile, with higher plasma clearance and higher steady-state volume of distribution (Table 1), and reduced adverse events compared to the standard AmB-DOC infusion in 5% dextrose [98,136,137,138]. A recent systematic review and network meta-analysis of 25 randomized controlled trials (RCTs) enrolling a total of 2996 patients, aiming to evaluate the efficacy and safety of conventional AmB and lipid formulations, identified AmB-IL as the safest cost-saving treatment [139].

Concerns due to lack of uniformity of AmB-IL admixtures led to the development of a preformed AmB-DOC fat emulsion, with submicron average particle size and the same composition of Intralipid^®^ 20% (mainly soybean oil 20% *w*/*v*, 1.2% *w*/*v* purified egg lecithin, and 2.25% *w*/*v* glycerin) [140,141]. Studies suggest that the strong interaction between AmB and oil droplets forms a reservoir of monomeric AmB, the less toxic form of the drug [140,141]. This standardized AmB O/W emulsion (ABLE, Amphomul^®^) is currently commercialized in India for the treatment of VL and febrile neutropenia in cancer patients [140,141]. Although clinical pharmacokinetic data is not available, studies in male New Zealand white rabbits showed a peak plasma concentration (*C*_max_) of 0.387 ± 0.176 μg/mL, an area under the concentration–time curve (AUC) of 1.115 ± 1.558 μg/h/mL, a half-life (*t*_1/2_) of 6.622 ± 10.63 h, a clearance (CL) time of 16.06 ± 12.5 mL/h/kg, and an apparent volume of distribution (*V*_d_) of 26.14 ± 18.52 L/kg after administration of ABLE as a single i.v. bolus dose (5 mg/kg body weight) [140]. Despite the low plasma peak levels, fast accumulation in the liver and spleen (the target organs in VL) due to RES uptake may contribute to increased efficacy and reduced toxicity [140]. In a phase 3 RCT (NCT00876824) to assess the efficacy and safety of a single 15 mg/kg Amphomul^®^ infusion in 376 patients with VL, nephrotoxicity and hepatotoxicity were not observed [141].

Compared to parenteral administration, pulmonary delivery of AmB for the treatment or prevention of lung fungal infections is an attractive strategy to minimize systemic exposure to the drug, avoid infusion-related side effects and increase AmB residence time at the site of infection [142]. Conventional AmB-DOC and commercial lipid formulations (L-AmB, ABLC, and ABCD) can be efficiently nebulized yielding aerosol particles with mass median aerodynamic diameter (MMAD) in the range 1–5 μm (similar to the size of fungal spores) suitable for inhalation [143]. Pulmonary deposition of AmB in healthy rats directly after nebulization of the aforementioned formulations achieved concentrations above the MIC of *A. fumigatus* and the drug was still detected in the rat lungs six weeks after nebulization [143]. In persistently granulocytopenic rats with invasive pulmonary aspergillosis, both prophylaxis and treatment with nebulized AmB-DOC or any of the aerosolized commercial lipid formulations at one week before or 16 h after fungal inoculation, respectively, resulted in significantly prolonged survival [143]. In a phase 3 open-label clinical trial (NCT00177684), administration of ABLC (Abelcet^®^) for four days via aerosolized nebulization in 48 lung transplant recipients with invasive aspergillosis resulted in therapeutic AmB concentrations in the epithelial lining fluid nearly 168 h after the last inhaled dose [144]. The liposome composition of L-AmB is similar to the lipid composition of endogenous pulmonary surfactant, and nebulized L-AmB (AmBisome^®^) has been commonly used in the clinic as a prophylaxis of lung fungal infections in immunocompromised patients [142,145,146] without changes in surfactant lipid composition or the deleterious effects of deoxycholate on lung surfactant function of inhaled AmB-DOC [147]. Compared to nebulization of liposomal solutions, dry powder formulations for inhalation manufactured by spray-drying provide proliposomes with improved stability that can be administered using portable dry powder inhaler devices, being converted to liposomes in situ by hydration upon contact with the aqueous milieu of the lung [148].

Patent expiration protecting the original lipid-based AmB formulations in the market represented an opportunity for the introduction of less expensive generics. However, generic manufacturing of AmB lipid formulations requires careful control of processing conditions and appropriate bioequivalence testing, since changes in phospholipid composition, size and charge of liposomes, drug–lipid molar ratio as well as the manufacturing process can alter the formulation efficacy and toxicity [25,149,150]. Even liposomal formulations with the same chemical composition of AmBisome^®^ that reached national markets, such as Phosome^®^, Lambin^®^ or Anfogen^®^, may reveal distinct pharmacokinetics, drug release and safety profiles, suggesting that different manufacturing processes may alter the properties of the final product [149,151,152,153]. Anfogen^®^, originally marketed in Argentina, exhibited higher red blood cell (RBC) hemolysis in vitro [151] and increased damage to kidney cells in vivo [151] compared to the parent formulation (AmBisome^®^); it was withdrawn for further development [149,151]. A steady-state global bioequivalence study comparing 50 mg/vial L-AmB generic injectables and reference AmBisome^®^ in VL patients under fed conditions (NCT03636659) was recently completed but results have not been published yet.

Nanotechnology may also provide promising solutions for the development of more efficient and safer drug delivery systems (DDSs) for AmB. A novel AmB liposomal formulation (L-AmB-LRC-1, Fungisome^TM^), with an optimal lipid to drug ratio developed in India, was introduced to the Indian market in 2003 for the treatment of systemic fungal infections and VL [25,42,105]. Fungisome^TM^ differs from AmBisome^®^ in liposome composition and manufacture (Table 1). The formulation is prepared with multilamellar vesicles (MLVs) stabilized in saline, which are more stable than small unilamellar vesicles (SUVs) at high temperatures typical of tropical and subtropical regions, thus withstanding longer storage times without loss of efficacy [105]. Although the formulation requires ultrasonication for 45 min prior to infusion in order to convert MLVs into small, uniform liposomes (nanosomes) with sizes in the range of 20–200 nm for improved biodistribution [105], a retrospective post-marketing surveillance documented the high therapeutic efficacy of Fungisome^TM^ at a lower dose (1–3 mg/kg/day) and minimal nephrotoxicity [154], representing a cost-effective alternative to AmBisome^®^ therapy. Fungisome^TM^ is also available as an AmB gel 0.1% *w*/*w* formulation for topical application in skin fungal infections and cutaneous leishmaniasis (CL).

Another nanosomal AmB formulation for injection was developed from soy phosphatidylcholine (SPC) and sodium cholesteryl sulfate employed as generally regarded as safe (GRAS) lipid excipients using an aqueous medium free of toxic organic solvents and detergents in the manufacturing process, which yielded a homogeneous population of nanosized particles below 100 nm [155]. Nanosomal AmB (Amfy^®^) was shown to provide a safe and cost-effective alternative to AmBisome^®^ for the treatment of fungal infections [155]. An AmB gel formulation for topical application was also prepared by mixing the nanosomal AmB lipid suspension with an aqueous solution of carbomer homopolymer [156]. The efficacy and safety of the lipid-based AmB gel 0.1% *w*/*w* for the treatment of recurrent cutaneous and/or mucocutaneous fungal infections were demonstrated in an open label clinical study enrolling 100 patients [156]. Both the parenteral and the topical nanosomal AmB formulations are currently marketed in India for the treatment of life-threatening systemic and (muco) cutaneous fungal infections, respectively.

## 4. Investigational Lipid-Based Systems for Amphotericin B Delivery

The development of a nanotechnology-based DDSs for AmB represents a promising approach to less toxic and equally or more effective antifungal therapies than conventional AmB-DOC. Nanoparticles (NPs) provide the opportunity for selective targeting of AmB to fungal cells and for sustained and controlled drug release, reducing the drug’s toxic side effects and improving its pharmacokinetic profile [157]. Moreover, nanoparticulate DDSs have the potential to overcome the poor water solubility of AmB and improve its membrane permeability and oral bioavailability.

Lipid vehicles are attractive AmB delivery systems due to the drug’s ability for binding lipids. However, commercially available AmB lipid formulations for the treatment of invasive fungal infections are expensive and require parenteral administration, increasing length of hospital stay and healthcare costs. Therefore, the development of an orally available AmB formulation able to decrease the systemic toxicity of the drug, avoid infusion-related adverse events, improve patient compliance, and reduce the costs associated with commercial AmB formulations for intravenous administration is an urgent requirement [41,158,159,160]. Lipid-based systems for AmB delivery which mainly developed over the last five years are summarized in Table 2 and will be further discussed in the next sections.

### 4.1. Lipid Conjugates

Lipid conjugation to AmB has the potential to reduce drug toxicity and increase its oral bioavailability by improving stability and absorption in the gastrointestinal tract (GIT). Oleic acid (OA), a known skin and intestinal permeation enhancer, has been conjugated to AmB via amide bond formation with the carboxylic acid group of the drug using standard carbodiimide chemistry [161]. Metabolism of the AmB-OA conjugate in liver homogenate was higher than 80% [162], which warrants prodrug bioconversion after oral administration. AmB-OA was stable in simulated gastric fluid (pH 1.2) and displayed enhanced permeation across the human colon adenocarcinoma (Caco-2) cell monolayer as an intestinal barrier model compared to the free drug [162]. Cytotoxicity concerns resulting from enhanced intestinal permeability were also evaluated and cell viability of Caco-2 monolayers upon exposure to AmB-OA for 3 h was found to be higher than 90% [162]. A reversible reduction in transepithelial electrical resistance (TEER) values was observed, indicating monolayer integrity retention. Oral administration of AmB-OA conjugate to rats (10 mg/kg in phosphate buffer saline (PBS) as gavage vehicle) resulted in significant increase in *C*_max_ and AUC compared to i.v. AmB and AmB-OA admixture [162].

Contrary to free AmB, the concentration-dependent aggregation of AmB-OA did not result in hemolytic toxicity or nephrotoxicity in vitro, which was attributed to differential aggregation behavior of AmB-OA [161]. The results were corroborated by in vivo studies in healthy mice after oral administration of AmB-OA (10 mg/kg in PBS) showing no significant increase in the levels of nephrotoxicity or hepatotoxicity biomarkers compared to control (vehicle-treated mice) despite AmB-OA conversion into the parent drug in the liver. These findings were also supported by histopathological tissue examination [161]. Further in silico studies showed that monomers in AmB-OA dimers accommodate in a head-to-head arrangement in contrast to head-to-tail arrangement in AmB dimers [161]. Moreover, AmB-OA in the aggregated state retained selectivity for ergosterol over cholesterol, which was lost in AmB aggregates [161], and in vitro antifungal activity of the parent drug was retained in the AmB-OA conjugate [162]. These results suggest that lipid conjugation can be a promising strategy for oral delivery of AmB.

AmB conjugation to a di-walled molecular umbrella constructed using spermidine (a biogenic polyamine) as the scaffold and cholic acid as the umbrella walls improved cellular selectivity of the drug [163]. AmB conjugated to the bile salt-based molecular umbrella retained in vitro antifungal activity but reduced hemolytic activity and cytotoxicity to kidney cells [163]. The ability of molecular umbrellas to cross lipid membranes by passive diffusion can be useful to improve transport across the blood–brain barrier (BBB) and increase drug concentration in the brain with therapeutic potential in brain fungal infections [163].

### 4.2. Micelles

Micelles are association colloids formed by spontaneous self-assembly of surfactants in solution once surfactant concentration reaches the critical micelle concentration (CMC). AmB nanomicellar aerosols using sodium deoxycholate sulfate (SDCS) as the lipid vehicle have been developed for pulmonary delivery of AmB [164,165]. AmB-SDCS dry powder at a drug/lipid molar ratio of 1:2, mimicking AmB-DOC commercial formulation Fungizone^®^, was prepared by freeze drying and reconstituted with distilled water for jet nebulization, producing an aerosol with mean diameter in the range of 1–5 μm suitable for inhaler use [164,165]. Compared to conventional AmB-DOC, AmB-SDCS showed significantly reduced cytotoxicity in vitro against RBCs [164], kidney [124,165,166], lung [164,165,166], and macrophage [164,165] cell lines, but improved in vitro antifungal [164,165] and antileishmanial [165] activity. Phagocytosis of AmB-SDCS has been observed in vitro by fluorescence microscopy in an alveolar macrophage cell line, suggesting the therapeutic potential of the aerosol formulation for the treatment of invasive pulmonary fungal infections by targeting alveolar macrophages [124]. Following intratracheal instillation of AmB-SDCS for seven days in rats (1.5 mg/kg/day), biodistribution and histopathology studies revealed higher therapeutic AmB concentrations in lungs with no evidence of renal or hepatic toxicity [166]. Molecular dynamics simulation suggested stabilization of the AmB-SDCS complex via intramolecular hydrogen bonding that presumably contributed to the delayed release of the drug and reduced toxicity of this formulation [165].

Lipid-bile salt mixed micellar systems have been used to improve solubility and membrane permeability of AmB in order to enhance its absorption from the GIT and improve drug oral bioavailability. Our research group has developed lipoamino acid (LAA)-based micelles as AmB delivery vehicles [167,168,214]. LAAs offer several advantages as DDS since they can be obtained by biotechnological procedures from natural renewable sources (proteinogenic amino acids and naturally-occurring fatty acids or their derivatives), being biocompatible, biodegradable, and environmentally-friendly vehicles [167,214]. Micelles made from an anionic dimeric (gemini) LAA derived from cysteine [167], as well as equimolar mixtures of this LAA and either sodium cholate or sodium deoxycholate [168], were able to solubilize AmB in its monomeric and less toxic form, under biomimetic conditions [168]. Both pure LAA micelles and LAA-bile salt mixed micelles showed in vitro antifungal activity against *C. albicans* comparable to that of Fungizone^®^ [168]. Furthermore, the DOC-containing solutions formed a shear-thinning gel at concentrations above 0.01 mol L^−1^, suggesting the viability of topical AmB delivery in mucocutaneous fungal infections [167].

### 4.3. Liposomes, Ethosomes, and Niosomes

Liposomes are concentric bi-layered structures formed by self-assembly of phospholipids in aqueous media. Made from phospholipids structurally similar to those found in cell membranes, liposomes are biocompatible, biodegradable, and non-immunogenic drug carriers able to encapsulate both hydrophobic and hydrophilic drugs in the lipid bilayer or the aqueous core, respectively [111,215]. Lipid composition, surface size, and charge, as well as the manufacturing process, significantly influence liposome properties [111,215]. Phospholipids commonly employed include unsaturated (or hydrogenated) phosphatidylcholines (PCs) from natural sources, such as egg or soybean PCs, and anionic phosphatidylglycerol, with long acyl chains for high gel to liquid crystalline phase transition temperature (above physiological temperature) [111,215]. Addition of cholesterol improves liposomal stability in serum and hinders premature drug leakage [111,215]. However, stability in the GIT is hampered by chemical and enzymatic hydrolysis of the ester bonds in the phospholipid bilayers and by the membrane detergent effect of bile salt surfactants. Nevertheless, liposomes incorporating vegetable ceramides (mainly glucosylceramides) for oral delivery of AmB have been reported with improved membrane stability in an artificial stomach-duodenum model [216].

After systemic administration, liposomes are taken up by the RES, which allows passive targeting to the mononuclear phagocytic system with utility in antifungal and antileishmanial infections [215]. Active targeting of AmB-loaded liposomes to macrophages and RES has been achieved by the incorporation of a polysaccharide ligand (hydrophobized alginate) producing surface-modified liposomes (SML) with enhanced efficacy in both promastigote and amastigote models of VL due to increased macrophage uptake [169]. AmB-loaded magnetic liposomes (AmB-MLP) for brain targeting, with sizes around 240 nm, have also been prepared from OA-modified superparamagnetic Fe_3_O_4_ nanoparticles to enhance the drug concentration in the brain in the presence of a magnetic field, which was successfully achieved in vivo after carotid artery administration to rats [170].

Although AmB liposomal formulations for parenteral administration, such as AmBisome^®^, usually improve drug solubility, bioavailability, and pharmacokinetic profile while simultaneously reducing adverse events due to the site-avoidance mechanism, their production cost is high. Stigmasterol, an abundant phytosterol, can replace the expensive current good manufacturing practice (cGMP)-grade cholesterol used in liposomal formulations required to avoid the risk of viral or prion contamination upon purification of cholesterol from animal sources [171]. Moreover, covalent coupling of stigmasterol hemisuccinate to both *sn*-1 and *sn*-2 positions of glycerophosphocholine (GPC) produces liposomes with improved plasma stability since sterol transfer from the lipid bilayer is hindered [171]. Among the 32 different distigmasteryl hemisuccinoyl-phosphatidylcholine (DSHemsPC)-based liposomal formulations prepared by Iman et al. [171], AmB-DSHemsPC-DMPC-DMPG at 1.0:1.25:5.0:1.5 molar ratio showed in vitro antifungal and antileishmanial activity comparable to that of AmBisome^®^ [171] as well as similar biodistribution after i.v. administration to healthy mice [172]. The stigmasterol-based AmB-loaded liposomes were less hemolytic than the cholesterol-based ones (AmBisome^®^) [171,172], presumably due to the improved stability of the former, where the sterol moiety is immobilized in the lipid bilayer, thus reducing drug leakage. In *L. major*-infected mouse models of early and established lesions, both liposomal formulations at 5 mg/kg multiple i.v. doses were able to significantly reduce the parasite load in the spleen but were less effective in the reduction of parasite load at the footpad [172]. The results suggest that liposomal formulations are better suited for the treatment of VL than for the cutaneous form of the disease.

Ultradeformable liposomes (UDL) aiming to improve transdermal delivery of AmB in cutaneous fungal and leishmaniasis infections were prepared by Perez et al. [173]. Maximum deformability was achieved using Tween 80 as an edge activator, and these transfersomes were able to incorporate AmB in its monomeric and less toxic form [173]. AmB-UDL showed in vitro antifungal activity against *C.* *albicans* and non-*C.* *albicans* strains and also antileishmanial activity against promastigote and amastigote forms of *Leishmania braziliensis* at concentrations not cytotoxic to mammalian keratinocytes and macrophages [173]. In vitro studies using human skin explants revealed deep AmB penetration upon 1 h of non-occlusive incubation with AmB-UDL, and 40 times higher drug accumulation in the skin when compared to AmBisome^®^ [173]. Similarly, nanoethosomes loaded with AmB 0.1% *w*/*w* and incorporated in Carbopol^®^ gel base displayed enhanced skin permeation and deposition compared to a marketed gel formulation of similar strength [174]. The presence of ethanol in ethosomes, which enhances the deformability of the vesicles and may also fluidize intercellular lipids in the skin stratum corneum (SC), is presumably responsible for the permeation enhancement. The nanoethogel formulation also increased antifungal activity against *C. albicans* and showed no skin irritation in vivo in the Draize test [174].

Another liposomal formulation devoid of cholesterol has been developed based on the higher affinity of AmB for ergosterol. Ergosterol-rich liposomes of mixed lamellarity consisting of AmB, PC, and ergosterol at 1.8:5:2 molar ratios (Kalsome^TM^10) were able to encapsulate a higher amount of AmB than AmBisome^®^ (0.2 mg/mg vs. 0.143 mg/mg total lipid), which can contribute to a lower price of the ergosterol formulation [175]. Moreover, since cholesterol is required for macrophage internalization and parasite survival, cholesterol-free drug carriers may provide more appropriate delivery vehicles for leishmaniosis therapy [176]. Kalsome^TM^10 was more effective than AmBisome^®^ against *Leishmania donovani* upon endocytosis by the host macrophages [177]. Mechanistic studies revealed that Kalsome^TM^10 induced apoptosis in both promastigotes and intracellular amastigotes but not in mammalian macrophages [177]. Treatment of *L. donovani* infected mice with Kalsome^TM^10 (7.5 mg/kg triple dose i.v.) resulted in 99% amastigote suppression after one week of treatment and complete parasite clearance after one month [176], with no damage to either liver or kidney [175,176]. Further studies showed that the decline in parasite load was accompanied by a shift from Th2-type to Th1-type response correlated with the immunomodulatory properties of AmB [175].

Liposomal formulations have also been optimized for topical delivery of AmB in vulvovaginal candidiasis. A dispersion of cationic liposomes, made from dioleoylphosphatidylethanolamine (DOPE), 1,2-dioleoyl-3-trimethylammonium propane (DOTAP), and cholesterol (molar ratio 4:5:1), in a Poloxamer-based thermosensitive gel enhanced AmB solubility, improved stability, and reduced the drug toxicity in vitro [178]. These gels were liquid at room temperature but solidified near physiological temperature [178]. Entrapment of liposomes in the gel matrix can increase their residence time within the vagina, promote controlled and sustained drug delivery, and prolong the shelf life of the formulation.

Niosomes, which are vesicle systems made of non-ionic surfactants (often terpenoids, polysorbates, Spans, and oxyethylenes) and cholesterol, are structurally and functionally similar to liposomes but more advantageous than the latter, since phospholipids used in liposome manufacture are more heat sensitive and can suffer oxidative degradation. As surfactants are easily derivatized leading to a higher adaptability of the vesicular structure, niosomes are more stable and require less production costs than liposomes, and are attracting wide interest [217,218].

Alssadi et al. evaluated the effects of AmB-loaded niosomes (in the aerosolized form) on skin, lung, and liver of rat models of leishmanial and invasive pulmonary aspergillosis infections [219]. Treatment with formulations composed of Tween 80/cholesterol noisomes encapsulating hydroxypropyl-γ-cyclodextrin/AmB led to a significant reduction in fungal lung burdens and to an expressive suppression of *L. donovani* liver parasite burdens [219]. Results pointed to the improvement of AmB delivery to lungs and liver from a aerosolized niosomal formulation, with minimal systemic display and toxicity [219].

Glycolipid biosurfactants produced from renewable energy resources are also attractive eco-friendly raw materials for the production of niosomes due to their easy biodegradation, although availability can be a determinant of their cost [217,218]. Recently, Haque et al. [179] managed to increase the production of sophorolipids (SL) by *Starmerella bombicola* using rice bran and cottonseed oil, which are low-cost renewable substrates. These compounds are known for their antimicrobial and antiadhesive properties, therefore AmB-SL niosomes may exhibit potential synergistic antifungal and antibiofilm effects [220]. Biofilm eradication concentration required to reduce pre-grown biofilm cell viability by 50% (BEC_50_) compared to untreated cells (control), determined after 24 h incubation of mature *C. albicans* biofilms with AmB-SL niosomes or AmB alone, was 0.195 μg/mL and 0.390 μg/mL, respectively [179]. A biphasic release pattern was observed for AmB-SL niosomes under physiological conditions (PBS, pH 7.4) which correlated with anti-biofilm activity [179]. Confocal microscopy revealed decreased viability of *C. albicans* mature biofilms upon treatment with AmB-SL niosomes and the absence of pseudohyphae, which were present in the biofilms treated with a marketed liposomal formulation (Phosome^®^) [179].

### 4.4. Nanoemulsions

Nanoemulsions (NEs) are formulation strategies that enhance the solubility and the bioavailability of AmB [180]. NEs are O/W dispersions having nanosized droplets with high surface area that obey the prerequisite for enhanced adherence to the fungal cell surface, besides providing benefits in terms of preparation, drug solubilization, and controlled release [181,182,183,184]. Surfactants and co-surfactants that absorb at the surface of oil droplets to decrease the interfacial tension are required to stabilize the NE, thus preventing aggregation and coalescence. The characteristics of NEs are affected by the composition, processing parameters, and pH of the aqueous continuous phase [181].

Based on a strategy that uses the pH-solubility profile of the drug to formulate AmB-loaded NEs, Caldeira et al. prepared NEs containing AmB and cholesterol and studied the effect of the cationic lipid stearylamine (Sta) on drug encapsulation efficiency (EE), cytotoxicity towards macrophages and in vitro antileishmania activity [180]. The stability of NEs containing AmB can be thus accomplished from the production of an ion pairing between AmB and Sta (with intrinsic antileishmanial activity) [180,221]. Sta concentration did not affect EE, which turned out to be approximately 100%, nor the stability of the nanoformulation. In addition to EE, stability studies have also shown that particle size, polidispersity index, and AmB content remain constant after 180 days. However, authors observed a decrease of EE value in the absence of cholesterol, which confirmed the decisive role of the co-surfactant in the retention of AmB as already observed in lipid vesicles and micelles [222,223]. To study the efficacy of these NEs in an eventual i.v. therapy of leishamaniasis, in vitro activity against intracellular amastigotes and in vitro cytotoxicity on J774 murine macrophages were evaluated. In the formulation with Sta, cytotoxicity increased with the cationic lipid concentration, resulting from the electrostatic interaction of Sta with anionic constituents of the cell membrane [180]. But when compared to conventional AmB, the NEs showed a lower cytotoxicity probably due to the observed reduction of the self-associated free drug in AmB lipid vehicles. Sta improved the in vitro antileishmanial efficacy of NEs, in agreement with literature data which proposed a synergistic contribution of Sta and AmB. On the other hand, NEs containing AmB appeared to be more selective against the parasite, when compared to conventional AmB. Overall, Sta contributed also to a higher efficacy and cytotoxicity of the nanoformulation. The promising results indicate that AmB-NEs may be a potential strategy in the i.v. treatment against leishmaniasis, which can be seen as an outstanding evolution from previous studies concerning dermal treatment of AmB-loaded NEs [180,221].

Topical delivery of AmB should offer advantages over i.v. or oral routes, in terms of patient compliance and AmB therapeutic performance [182,183] despite the distinct physicochemical nature of AmB that compromises its skin permeation. Permeability enhancers such as dimethyl sulfoxide (DMSO) and cyclodextrins (CDs) have been added to AmB formulations to improve drug solubility [224]. Semisolid AmB topical formulations with added γ-CD showed shear-thinning and thixotropic behavior, improved skin permeability, and enhanced in vitro antifungal efficacy against *Candida* species and *Sacharomyces cerevisiae* compared with an AmB reference formulation containing no solubility enhancers [224]. Several studies on NE development for topical AmB delivery have shown their suitability for the treatment of skin fungal infections [180,181,182,183,184], based on the synergistic effects resulting from the combination of lipids and surfactants with the antifungal drug [181]. Hussein and co-workers evaluated the enhanced stability and permeation capacity of AmB-loaded NEs at varying pH, storage temperature, and skin permeation of AmB [181]. Sefsol-218 was successfully used as the oil phase, disrupting the crystalline lipid packing layer of SC, and Tween 80 and poly(ethylene glycol) (PEG)-400 were added as surfactant and co-surfactant, respectively [181,182]. Optimized formulation was prepared at pH 7.4 of NE aqueous phase, corresponding to the slightest degradation observed for the drug at room temperature, and presenting unaggregated non-toxic monomeric AmB. Permeation rate for the optimized AmB-NE was higher when compared to the commercial cream Fungisome™ and also to the NE formulated at pH 6.8 (which could be a suitable formulation due to its permeation flux rate, penetration capacity, and low irritant property for atopic dermatitis application). The addition of a co-surfactant may have contributed to the decrease of interfacial tension by enhancing penetration of the oil phase in the hydrophobic region of the surfactant monomers. Moreover, DMSO used in combination with Sefsol-218 in the oil phase as a penetration enhancer, promoted higher AmB permeation from the NE. Drug accumulation into albino rat skin was approximately two-fold higher than the Fungisome™ cream, in line with the higher permeation rate. Distinct drug permeation mechanisms concerning the commercial product and the developed NE could explain this observation. In the NE formulation containing the lipophilic fluorescence marker Rodhamine 123, in vivo skin studies indicated that the dye distribution depended on the Sefsol-218 content, showing an enhanced release of AmB into the deeper area of the rat skin, contrary to what was observed in the commercial cream. Results from the rheological evaluation suggested a viscosity modification in order to make AmB-NE suitable for topical delivery. Furthermore, the shelf-life of the optimized formulation was found to be considerably higher when stored at 5 °C as compared to 40 °C, and 99.3% of AmB remained undecomposed in the vehicle at 5 °C against 87.1% at 40 °C.

In another study, Hussein and collaborators developed AmB-NEs using lipid Capmul PG8 (CPG8) instead of Sefsol-218, surfactant Labrasol (LAB) with inherent antifungal capacity, and co-surfactant PEG-400 [182]. The in vitro drug release behavior of the optimized AmB-NE showed a lower percentage of the drug released within 60 min when compared to the drug in DMSO, implying interactions of AmB with the dialysis membrane. Nevertheless, in comparison with Fungisome™, AmB-NE showed a higher drug release over a period of 12 h. AmB-NE exhibited a slow and sustained release pattern of the drug perhaps due to its viscosity and low partitioning of the drug from the nanoglobules to the buffer medium. Permeation flux of AmB-NE and the percentage of drug deposited across abdominal albino rat skin after 24 h were higher as compared to the commercial formulation, due to the role of LAB and PEG-400 as permeation promoters. The significantly enhanced antifungal activity of AmB-NE was mirrored in the higher MIC values against *C. albican*s and *A. niger*, as compared to the free AmB. In vivo penetration study across the rat skin layer confirmed the ex vivo permeation findings. As observed before [181], drug penetration into the epidermis and dermis of rat skin was augmented in the case of NE. Surface activity of LAB and CPG8 and the nanosize of the carrier appear to play a significant role in this enhanced penetration [181,182].

More recently, Hussein et al. proposed the use of NEs for topical delivery of AmB using as major components the oily vehicle Peceol and surfactant Labrasol (LAB), which have innate antifungal potential probably due to the presence of capric and caprylic acid in their chemical composition [183]. An eventual toxicity by surfactant was minimized with the addition of co-surfactant propylene glycol (PG) which lowered the surfactant/co-surfactant mixture (S_mix_) concentration in the final formulation [182,183]. AmB-NE optimized formulation has been shown to significantly attenuate in vitro hemolysis, which predicted a lower in vivo hemolysis by reaching systemic circulation due to its smaller size [183]. Enhanced ex vivo abdominal rat skin permeation compared to AmB-DOC seems to result from the particle size decrease, also due to NEs’ ability to lower interfacial tension between the carrier and the skin. Moreover, PG and LAB enhanced permeation by possibly extracting the lipids of the SC layer, breaking the lipophilic barrier. The partial involvement of the surfactants in the solubilization of AmB may be the reason for the reduced perturbation in the SC exhibited by AmB-NE. The augmented permeation flux observed for the optimized NE formulation, as compared with AmB-DOC, may have led to the high drug deposition of the optimized nanoformulation. Increased antifungal activity was observed in the in vivo evaluation against *A. fumigatus* and *C. albicans*, indicating a more destructive effect of AmB loaded into NE [183]. Thus, the nanocarrier developed by Hussein et al. showing enhanced therapeutic efficiency and high stability, may be indicated for safe and effective use in topical applications to treat skin fungal infections [183].

Sosa et al. also developed an AmB-NE formulation based on castor oil, Labrasol^®,^ and Plurol^®^ oleique (surfactant/co-surfactant mixture) and Transcutol^®^ P, chosen according to their ability for skin delivery and for drug solubilization [184]. Newtonian behavior exhibited by the optimized AmB-NE indicated its suitability for topical application, namely as a spray or roll-on. Results from the in vitro release study showed that 100% of AmB was released from NE after 75 h following the one-step sustained release pattern, contrary to the very low release rate of AmB plain solution (below 10%). Skin integrity assessment (through in vivo tolerance study performed in humans) showed that the nanoformulation did not change the biophysical properties of skin, being appropriate and safe for topical application. Based on the skin retention and MIC values, and considering the density of hydrated skin, the AmB-NE developed by Sosa et al. appears to provide an efficient local delivery of the drug without theoretical systemic absorption, and possibly without the detrimental side effects. These benefits would be considerably useful not only in treating fungal skin infections but also against fungus presented in deep layers [184]. However, according to permeation studies carried out by Sosa et al. in human skin, no systemic concentration of AmB is expected to occur, in opposition to the results obtained by Hussein and co-workers in rat skin [182,183,184].

### 4.5. Self-Emulsifying Drug Delivery Systems

Self-emulsifying drug delivery systems (SEDDSs) are isotropic mixtures of oils/lipids, surfactants, solvents, and co-solvents/surfactants designed to improve the oral bioavailability of poorly water-soluble drugs, enabling the drug for other non-invasive routes as well. Emulsifying conditions of the preparation process mimic those of the GIT. In addition, SEDDSs are able to emulsify on body tissues having some wet dispersion medium, such as ocular, buccal, nasal, and vaginal mucosa. Based on droplet size, SEDDS formulations can be named self-micro-emulsifying drug delivery systems (SMEDDSs, droplet size 100–250 nm) and self-nano-emulsifying drug delivery systems (SNEDDSs, droplet size below 100 nm).

SEDDS formulation iCo-010 (recently completed Phase I clinical trials) developed by Wasan et al., and containing monoglycerides, diglycerides, polyethylene glycol glycerides, and D-α-tocopheryl polyethylene glycol succinate (TPGS), proved to be efficient in the treatment of fungal and leishmaniasis infections [185,186,187,225,226]. The enhanced oral absorption of iCo-010 can be related to the solubilization of AmB dispersion within the emulsion particles following its mixing with GI fluids [226]. The increase of intestinal wall permeability and the prolongation of GI transit time can also promote the increase of oral absorption of the drug from the SEDDS, being hypothesized by the authors as decisive factors contributing to the required efficacy of iCo-010, in addition to the capacity of the formulation to target the lymphatic transport system [41,226]. In fact, along with favorable self-emulsifying properties and optimal stability, this SEDDS formulation exhibited a high antileishmanial activity in a murine model of VL [41,186], as well as in an acute model of systemic candidiasis in rats and in a mouse model of chronic systemic candidiasis infection [41,188].

Furthermore, no GI toxicity, hepatotoxicity, and nephrotoxicity were observed after multiple oral administrations of iCo-010 in the mouse model [225]. Nevertheless, the low level of the drug solubilized in the aqueous fraction (below 20%) during lyposis studies (and indicating its availability for absorption), is one the limitations of this formulation [226]. The biodistribution pattern revealing an uptake in the organs of RES at levels above the IC_50_ for the *Leishmania* parasite, supported the efficacy results and drove the formulation into phase I clinical trials [41,225]. Primary safety and tolerability endpoints were met in the phase I clinical trials of iCo-010, known actually as iCo-019 [41], and stability data supporting a period of 2.5 years have already been achieved [227]. A multi-ascending dose (MAD) study is expected to begin in healthy subjects [227].

Khan and co-workers developed a SNEDDS for topical and oral administration of AmB in leishmaniasis [189]. Authors prepared two formulations, formulation A (FA) and formulation B (FB), by mixing different excipients and using Tween 80 as a permeation enhancer in FB. Toxicity studies on Caco-2 cell lines assured the favorable safety profile in terms of oral and topical routes. A mucus permeation study, carried out on porcine GIT, indicated that both formulations were able to travel up to 8 mm of mucus. Permeation could be explained in terms of size and negative zeta potential values of FA and FB droplets. In vitro transport of SNEDD formulations across the Caco-2 cells showed that the percentage of AmB transport was faster in the first hour, which is a good property to avoid the loss of AmB from GIT. During the total time period of the permeation study, the integrity of the cell monolayers was preserved. The spreading efficiency of these nanoformulations was evaluated over buccal mucosa and an ulcerated skin model. The remarkable results proved that SNEDD formulation of AmB can disperse and spread on damaged tissue providing the drug to adjoining ulcerated tissues [189]. The IC_50_ values of FA and FB formulations against both promastigote and amastigote cultures of *L. tropica* (and also against the macrophage harbored stage) are significantly lower than the conventional AmB formulation. Overall, the outcomes of this study highlighted the potential of the SEDDS strategy to safely deliver AmB through oral and topical routes, useful for both VL and CL [189].

### 4.6. Cubosomes

Lipid-based cubic liquid crystalline nanoparticles (Cubosome^®^) have been explored as sustained-release DDSs to improve oral bioavailability of poorly water soluble drugs [228]. Glyceryl monooleate (GMO) and phytantriol (PHY) are common oily excipients with similar phase behavior able to form bicontinuous reverse cubic phase lyotropic liquid crystalline nanostructures in excess water at room temperature [228]. Cubosomes are conventionally prepared by fragmentation of a bulk lipid melt containing added poloxamer or other nonionic surfactant for stabilization and dispersion in water, usually with high-pressure homogenization [228]. However, due to AmB poor solubility in both aqueous and oil phases leading to low EE, other methods have been developed that use hydrotropes (e.g., methanol) [190] or O/W emulsions prepared by dissolving AmB-DOC micellar dispersion in a parenteral emulsion, such as Intralipid^®^ or Lipofundin^®^ [191,229].

Both GMO and PHY cubosomal formulations have been evaluated for oral delivery of AmB [190,191,192]. X-ray diffraction studies of freeze-dried samples showed the presence of encapsulated AmB in the amorphous form while UV-Vis spectroscopy of the colloidal dispersions revealed the incorporation of AmB predominantly in the monomeric form [190]. Compared to GMO, which can be cleaved by chemical or enzymatic hydrolysis in GI fluids due to its ester structure, PHY is a non-digestible terpenoid alcohol with a saturated diterpene chain, extensively retained in the stomach [228]. Persistence of the liquid crystalline nanostructure in the GIT is crucial for sustained drug release from cubosomes and a link between digestibility, gastric retention, and a sustained release effect has been established [228].

AmB-GMO and AmB-PHY cubosomal formulations were stable in simulated gastric and intestinal fluids [190,191,192] and showed increased uptake by Caco-2 cells via both clathrin- and caveolae-dependent transport mechanisms [192]. Reversible decrease in TEER measurements was observed and monolayer integrity was confirmed by actin visualization [190]. In vitro release profile in PBS buffer (pH 7.4, 37 °C) showed sustained drug release with slower release from AmB-loaded PHY cubosomes and absence of burst effect [190]. Pharmacokinetic studies in rats showed a significant increase in oral bioavailability of the cubosomal formulations compared to the free drug, in the order AmB-PHY > AmB-GMO >> AmB, according to the increased stability of acid-resistant PHY in the GIT [190]. In a rat model of systemic candidiasis, oral gavage administration of the AmB cubosomal formulation resulted in reduced colony counts in the kidney, but not in the spleen, liver, and lung, suggesting preferential distribution of the drug into the kidneys [192]. This aspect raises toxicity concerns, despite no signs of nephrotoxicity according to serum creatinine and blood urea nitrogen (BUN) levels [191].

Biodegradable cubic gel delivery systems that can function as drug depots for long-term sustained release have also been developed from bulk GMO cubic liquid crystalline phase for intra-articular administration of AmB in the treatment of fungal arthritis [193]. Due to high viscosity, these gels have low syringeability and are generated in situ from injectable GMO hydrolipid formulations upon contact with synovial fluid after intra-articular administration [193]. These formulations usually contain added hyaluronic acid as viscoelastic scaffold and co-solvents, such as ethanol and propylene glycol, to decrease the viscosity of the system and improve syringeability [193]. An AmB 0.1% *w*/*w* formulation consisting of GMO 55% *w*/*w*, water 15% *w*/*w*, soybean oil 5% *w*/*w*, and the aforementioned additives was found to provide long-term sustained release of AmB after intra-articular administration to rabbits, without symptoms of inflammation at the injected joint [193].

### 4.7. Cochleates

Cochleates are spiral multilayered structures made of solid lipid bilayer sheets with almost no internal aqueous space formed by precipitation of negatively charged phospholipids, such as phosphatidylserine, in the presence of a divalent cation, usually calcium [230]. Hydrophobic drugs like AmB are internalized within the lipid bilayers of the cochleate structure and effectively protected from the harsh environment of the GIT [230]. The high tension at the bilayer edges of cochleates contributes to their membrane fusion ability promoting endocytosis [230]. Cochleates are actively taken up by macrophages and once within the low calcium environment of the cytoplasm, the cochleate structure is no longer stabilized, releasing the drug, while the macrophage travels to the site of infection driven by humoral response [230]. This avoids systemic exposure to the drug and results in decreased toxicity, turning cochleates into versatile vehicles for oral, parenteral or topical drug delivery.

Cochleated AmB (CAmB), an oral formulation developed using proprietary lipid nano-crystal (LNC) delivery technology platform, has shown in vitro and in vivo antifungal efficacy [230,231]. In a mouse model of systemic candidiasis, all mice treated with oral CAmB for 15 days (0.5–20 mg/kg/day) survived the experimental period and a dose-dependent reduction of fungal burden was observed in lungs and kidney [194]. Oral CAmB at 2.5 mg/kg/day suppressed lung fungal load and was comparable to intraperitoneal (i.p.) Fungizone^®^ at similar dose and 10 times more effective than oral AmBisome^®^ [231]. Similarly, a survival rate of 70% was obtained in a murine model of aspergillosis after 14-day treatment with oral CAmB (20 and 40 mg/kg/day) accompanied by at least 2-log reduction in colony counts in lung, liver, and kidney [232]. Histopathology analysis revealed no signs of damage to target organs due to orally administered CAmB [231]. CAmB has also shown in vitro activity against *Leishmania chagasi* (ED_50_ 0.017 μg/mL) in a macrophage model of infection, at concentrations not toxic to the macrophages [233].

More recently, oral CAmB, in combination with flucytosine, was shown to be superior to oral fluconazole in a mouse model of cryptococcal meningoencephalitis, without significant adverse events [195]. Brain transport of fluorescent-labeled CAmB and enhanced drug concentrations in the brain were demonstrated from fluorescence measurements in treated mice [195]. Oral CamB (25 mg/kg/day) and parenteral AmB-DOC (5 mg/kg/day i.p.), combined with oral flucytosine (250 mg/kg/day), showed equivalent efficacy and produced similar immunological profiles in mice after three-week treatment [195]. The FDA has designated CAmB as a Qualified Infectious Disease Product (QIDP) with fast track status for the prophylaxis of invasive fungal infections due to immunosuppressive therapy and treatment of invasive candidiasis, invasive aspergillosis, and cryptococcal meningitis, and very recently granted orphan drug designation to the product for the treatment of cryptococcosis [234]. CAmB (MAT2203) is currently in phase II clinical development.

A novel cochleate formulation containing detoxified LPS from *Neisseria meningitides* B as a pathogen-associated molecular pattern for immunomodulating properties has recently been developed for the treatment of sporotrichosis [196]. Adjuvant Finlay Cochleate 3 (AFCo3), acting as a vaccine adjuvant and drug delivery agent for AmB, has been evaluated for its immunomodulatory and antifungal activities against *Sporothrix schenckii* [196]. Compared to the free drug, AFCo3-AmB showed enhanced antifungal efficacy in vitro and reduced cytotoxicity against peritoneal macrophages and murine erythrocytes. This formulation showed improved fungicidal activity against intracellular yeast in peritoneal macrophages and stimulated ex vivo release of pro-inflammatory mediators from splenocytes [196]. In vivo, AFCo3-AmB was also more effective than the native drug at reducing spleen and liver fungal load after i.p. administration (5 mg/kg/day for 5 days) in a mouse model of systemic infection, which was accompanied by a significant induction of Th1/Th17 response [196]. No significant changes were observed in BUN and creatinine plasma levels in mice treated with AFCo3-AmB compared with control group (untreated animals), suggesting no nephrotoxic effects at the tested doses since efficient targeted delivery of AmB to macrophages due to the presence of LPS in the cochleate structure reduces systemic exposure to the drug [196].

### 4.8. Nanodisks

Nanodisks are non-covalent structures composed of a phospholipid bilayer and a scaffold protein, usually apolipoprotein A-I (ApoA-I) or modified versions [197]. Pioneer studies by Burgess et al. pointed to favorable antifungal effects and tolerability of AmB nanodisks [197]. Based on the fact that the aggregation state of AmB influences the cytotoxicity for mammalian cells, Burgess and co-workers developed a nanodisk (ND) delivery system comprising a phospholipid bilayer, a scaffold protein engineered from apolipoprotein A-I, and super-aggregated AmB (AmB-ND) [197]. Results from studies performed in mice evidenced a better tolerability compared to AmB-DOC and L-AmB formulations, while preserving the characteristic antifungal efficacy of AmB-DOC [197].

Cole et al. investigated the efficacy and toxicity of AmB-ND after i.v. administration to different mouse models, susceptible and resistant to *L. major* [235]. During the experimental period, susceptible Balb/c mice treated with AmB-ND showed minimal alteration in footpad thickness, in opposition to animals treated with empty ND. Regarding the effect of AmB-ND on resistant strains in CH3 mice infected with *L. major*, no change in feet thickness and no footpad ulcerations were observed [235]. Considering both mouse models, AmB-ND managed to reduce the harshness of footpad lesions, although the therapeutic effects were more noticeable in the susceptible strain than in the resistant CH3 strains. No renal toxicity nor other evidence of toxicity were observed considering the dosage, route of administration, and treatment regimen followed by the authors. Furthermore, a prolonged and enduring therapeutic effect was verified [235].

Cho and co-workers studied the efficacy and safety of AmB-ND for sinonasal delivery using an in vitro model, based on the assumption that the nasal epithelium would be minimally damaged by AmB formulated inside the ND carrier while maintaining the same antifungal power as in the commercial AmB lipid formulations [198]. The reduction of lactate dehydrogenase (LDH) release (almost 85%) in epithelial cells exposed to ND-AmB formulation assured its ability to protect human nasal epithelia membranes without compromising antifungal activity, suggesting a potential role for ND-AmB in the topical AmB delivery into the sinonasal epithelium [198]. In accordance with previous findings [197], results also showed the ability of AmB-ND formulation against *A. fumigatus* equivalent to commercially available AmB, which may be indicative for its effective use in treating fungal rhinosinusitis (FRS). Furthermore, this potential therapeutic approach may be of great relevance in FRS considering the current absence of clinically effective topical antifungal therapies, especially in immunocompromised patients with invasive fungal sinusitis [198]. In vivo studies are thus required to evaluate the efficacy and the safety of a topical ND-AmB formulation in animal models, and to determine the effective concentration of AmB as well [198].

### 4.9. Solid Lipid Nanoparticles and Nanostructured Lipid Carriers

Solid lipid nanoparticles (SLNs) are nanosized spherical shaped structures composed of a solid lipid core stabilized by surfactants and eventually co-surfactants [157,199,202,236,237]. Several lipids are used in the formulation of SLNs, such as triglycerides (tricaprin, trilaurin, tripalmitin), hard fat type lipids (glycerol behenate, glycerol palmitostearate), and waxes (ethyl palmitate). Phospholipids, bile salts, polyvinyl alcohol, polyoxyethylene ethers, and polyethoxylated sorbitan esters are often used as biocompatible emulsifying agents. When compared to other DDSs, SLNs present many advantages such as a higher physical stability, the possibility to incorporate both hydrophilic and lipophilic drugs, better biocompatibility and lower biotoxicity, and a facilitated scale-up [199,236]. Moreover, SLN size and liposolubility allow drug diffusion through some biological barriers like the BBB, and they are not easily taken up by cells of the RES, which reduces their accumulation in the liver or spleen [236]. SLNs were shown to be endocytosed by macrophages and their improved antifungal activity may be related to the modified tissue distribution and macrophage loading [238].

Matrix type and AmB location in SLNs determine drug release from the formulation [236]. Aiming to determine the location of AmB in the lipid matrix, Tan et al. formulated and optimized an AmB-SLN DDS for oral administration involving a composite matrix of bee’s wax and theobroma oil. The dispersion pattern observed by the authors confirmed that the drug was evenly dispersed within the lipid matrix, contributing to a delayed gastric residence time and a slow oral AmB release [239].

Chaudhari et al. prepared SLNs containing supper-aggregated AmB (AmbiOnp) for oral administration that accumulated to a lesser extent in the kidneys [200,201]. Results from in vivo distribution studies (confirmed by renal toxicity studies) in rats showed that oral AmbOnp exhibited a better safety profile as compared to conventional i.v. administered Fungizone^®^. No adverse reactions were reported by the authors, as single-dose administration of the formulations was well tolerated. The nanoparticulate formulation showed improved relative bioavailability and reduced nephrotoxicity in comparison with Fungizone^®^ according to the in vivo pharmacokinetics studies. These results evidenced the high sustained release of AmB from the SLN formulation over a longer duration of time which could be ascribed to the slower release of entrapped AmB from the nanoparticulate matrix. The 60% drop in fed state simulated intestinal fluid observed may be related to the presence of high molar concentration of lipids bile salt and lecithin (illustrative of fats in foods) which promote the release of AmB from the nanoparticulate matrix in the course of their interaction with SLNs, suggesting that food could influence the oral bioavailability of AmB from SLN formulation [201]. In vitro AmBiOnp inhibitory activity against *C. albicans* indicated an increase in MIC value eight-fold higher than that of Fungizone^®^, imputed to the controlled release of AmB from the nanoparticulate platform. Together with the structural organization, high entrapment efficiency and particle size, the preparation technique may also have contributed to a selective self-association of the drug in the form of nontoxic water-insoluble super aggregates, leading to an augmented oral bioavailability and superior safety profile, and making AmBiOnp a promising alternative to the current i.v. therapeutic approach [200,201].

The improved bioavailability observed with oral delivery of SLNs could be related to an increase of residence time within the gut due to the interactions of lipids on the surface of NPs with the epithelial membranes [160]. Gastric and intestinal enzymes are prone to degrade lipid-based delivery systems and absorption can be enhanced in the course of the digestion of triglycerides from SLNs by pancreatic lipase [239,240]. Stability, drug release behavior, and in vivo performance of SLNs are also related to the location of the drug in the lipid matrix. Tan et al. formulated and optimized an AmB-SLN DDS for oral administration involving a composite matrix of bee’s wax and theobroma oil. The dispersion pattern observed by the authors confirmed that the drug was evenly dispersed within the lipid matrix, contributing to a delayed gastric residence time and a slow oral AmB release [239].

Moreover, SLNs tend to suffer aggregation as they have a large surface area, which eventually causes the decrease of the particle interaction with the intestinal mucosa. The results obtained by Amekyeh et al. indicated a possible aggregation of SLNs in the stomach with particles showing an optimal size (below 350 nm) and surface charge for absorption in the small intestine [241]. Food status may affect the bioavailability of SLNs containing AmB, resulting from changes in the drug dissolution prior to absorption, changes in GI residence time of formulation or alterations in drug membrane permeability, and absorption [240]. The effect of food on the GIT of AmB-loaded SLNs was studied in rats, using paracetamol and sulfapyridine as marker drugs to evaluate gastric emptying and cecal arriving, respectively [240,242]. The obtained data revealed that rate absorption of AmB from SLNs decreased with the presence of food, contrary to the extent of absorption that remained practically unchanged [240]. Regardless of the food status, these results suggested that amphotericin SLNs could be mainly taken up by the lymph, with the small intestine as the best delivery site for the prepared nanoformulation [240].

AmB containing SLNs for topical usage were designed and developed by Butani and co-workers, in an attempt to improve the penetration of AmB into the skin [199]. In vitro antifungal activity against *Trichophyton rubrum* indicated a higher efficacy for the optimized AmB-loaded SLN, the highest uptake of AmB in rat skin after ex vivo skin permeation evaluation, better retention in skin, and reduced skin irritation when compared to conventional AmB formulation [199]. Results pointed to an increase of AmB permeation through the skin led by lipidic NPs in accordance with literature reports indicating an enhancement of dermal delivery due to the similarity between SLNs composition and that of subcutaneous lipids [199]. The disruption of the tightly packed lipids that fill the extracellular spaces of the subcutaneous layer seems to be the mechanism by which nanoparticulate lipids increase skin permeability. All the results confirmed the advantages of SLNs as convenient systems for carrying poorly aqueous soluble AmB and for enhancing the therapeutic efficacy of the drug in topical applications.

A better anti-leishmanial efficacy was achieved by Gupta et al. for AmB formulated in tristearin-based SLNs, employing the macrophage-specific ligand *O*-palmytoyl mannan to modify the particles surface [238]. Compared to the unmodified formulation and AmB-DOC, the coated SLNs showed a higher antifungal activity after in vitro evaluation against *L*. *donovani* infected macrophage-amastigote system, which could be explained by the favored AmB delivery to macrophages through mannose receptors. Compared to AmB-DOC, a higher accumulation of coated AmB-SLNs in macrophage-rich organs (namely liver, spleen, and lungs) was observed in distribution studies carried out in male albino rats. The rate and degree of biodistribution seemed to be affected by the surface ligand anchoring. Furthermore, in vivo study against *L. donovani* infected hamsters also evidenced a better anti-leishmanial activity of coated AmB-SLNs [238].

Nanostructured lipid carriers (NLCs) are a second generation of SLNs with an oil core made of blends of spatially incompatible solid and liquid lipids developed in order to circumvent common disadvantages observed with SLNs, namely lipid crystallinity and polymorphic transition, lipid particle growth, and predisposition to gelation [157,202,236]. Due to their oily core and larger distance between the fatty acid chains, NLCs have increased drug loading capacity and reduced burst release effect compared to SLNs.

Santigo et al. prepared AmB-loaded NLCs (AmB-NLC) using glyceryl monostearate (GSM) and sesame oil [202]. Entrapment rate and drug release are influenced by the solubility of the drug in the lipid(s) that forms the internal phase of the NLC matrix. The selection of components of the lipid matrix is affected by the tendency of the liquid lipid to form crystalline structures. The crystallization process was expected to be slower for sesame oil when compared with Miglyol^®^ and sunflower oil, due to the presence of unsaturated and long-chain fatty acids. Furthermore, crystal formation rate and the polymorphic transitions of the lipids in the NLC structure may affect the physical characteristics, stability, EE, and drug release of the nanoparticles. Sesame oil provided the smallest particles when used in a binary mixture with GSM showing a homogeneous particle size distribution. Pluronic^®^ F68 was employed to stabilize the NLC; the non-ionic surfactant prevents the adhesion of lipase on the surface of the dispersed phase, thus inhibiting the in vivo degradation of the lipid matrix.

In order to avoid precipitate formation, lyophilization was necessary to improve stability and shelf-life [202]. Diffraction patterns displayed by the lyophilized formulations (LYO-NLC and LYO-AmB-NLC) showed that AmB was fully solubilized in the crystalline lipid matrix, which was corroborated by thermal analysis. In vitro release profile of AmB from the NLCs showed a low AmB release rate for 72 h, which may be the result of AmB internalization in the lipid matrix, as indicated by the lack of significant differences between NLCs and AmB-NLCs regarding physicochemical characteristics and stability. The high EE and the slow drug release for AmB-NLCs confirmed the compatibility of AmB with the lipid matrix, in opposition to results obtained by Tan et al. indicating high values of AmB expulsion rates from SLNs [243]. A high rate of AmB encapsulation was inferred from the efficiency encapsulation evaluation, probably due to the core/shell structure of NLCs and the use of liquid lipid that allows preservation of AmB inside the nanocapsule [202]. The developed AmB-NLCs could be considered an option for AmB delivery, and further and specific assays are needed to assess the therapeutic potential of this promising AmB-loaded system [202].

AmB-loaded NLCs prepared from Sta and OA were developed for pulmonary delivery yielding spray-dried microparticles suitable for dry powder inhaler [203]. This formulation provided sustained drug release (88.2% up to 40 h), achieving localized action in the lungs, and reduced drug nephrotoxic side effects after pulmonary administration to rats, according to pharmacokinetic parameters, organ distribution studies, histopathology, and hematological data [203].

### 4.10. Lipid–Polymer Hybrid Nanoparticles

Lipid–polymer hybrid systems have been developed to overcome the problems arising from some lipid-based nanoparticulate carriers, such as liposomes and surfactant micelles, based on a combinatorial approach that takes advantage of the synergistic effect between a lipid and a polymer. Micelles formed by spontaneous self-assembly of synthetic or natural surfactants are dynamic structures and upon parenteral delivery may disintegrate due to plasma dilution, releasing their cargo before reaching the target [215]. Therefore, lipid–polymer hybrid micelles have been prepared using poly(ethylene glycol) (PEG), an FDA-approved biocompatible and hydrophilic polymer [215]. PEGylation has been shown to prolong blood circulation of colloidal DDS by producing nanosized micelles with a hydrophilic outer shell able to escape RES uptake [215]. Alvarez et al., using FDA-approved excipients, reformulated Fungizone^®^ by reconstitution with PEG-distearoylphosphatidyl ethanolamine (DSPE) micelles in 0.9% sodium chloride in order to simultaneously deaggregate AmB and deliver sodium supplementation for reduction of drug-related nephrotoxicity [204]. Deaggregated AmB-DOC showed reduced hemolytic activity in vitro and renal toxicity in vivo compared to the parent formulation while retaining similar in vitro fungicidal activity [204].

Chen et al. studied self-assembling AmB-loaded mixed micelles based on a combination of lecithin with commercial amphiphilic polymers (Pluronic, Kolliphor, TPGS, and DSPE-*N*-methoxy-PEG2k) [205]. Among the developed micellar systems, AmB-loaded micelles composed of lecithin and DSPE-PEG2k (Ambicelles) showed the best results in improving AmB solubility. Ambicelles exhibited increased parenteral as well as oral bioavailability in rats compared with Fungizone^®^ and reduced in vitro cytotoxicity [205].

Song et al. prepared linolenic acid (LNA)-modified methoxy PEG-oligochitosan conjugate micelles (MPEG-CS-LNA) for AmB encapsulation achieving more than 80% EE and retarding drug release [206]. The AmB-loaded hybrid micelles improved AmB solubility and decreased hemolytic activity and renal toxicity of the drug without affecting antifungal activity [206]. Enhanced fungal cellular uptake was observed, which was attributed to a combined inducement of LNA and oligochitosan [206].

A novel AmB hybrid NE was developed by Ishaq et al. using canola oil, hydroxypropyl methylcellulose (HPMC), Carbopol^®^, and Tween 80 [207]. The nanoparticulate formulation was effective against *Aspergillus* spp. and *Fusarium solani*, and also exhibited a pronounced in vitro activity against *L. tropica* at low concentrations. Being formulated with readily available excipients, this NE showed great potential for large-scale production as a cost-effective treatment of topical fungal infections and post-kala-azar dermal leishmaniasis (PKDL) [207].

Asthana and co-workers [208] formulated cationic Sta lipid–polymer hybrid nanoparticles (LPNPs) which presented characteristics of polymeric NPs and liposomes that allowed high EE, sustained drug release profile, and good tolerability [208]. The results evidenced high antifungal efficacy in vitro against intracellular amastigotes of *L. donovani*, considering the high uptake of LPNPs by macrophages, the rapidity of plasma clearance, and the significant drug allocation in liver and spleen macrophages [208]. In vivo results illustrated the high inhibitory activity against *L. donovani* amastigotes of infected mice. A very positive safety profile based on a reduced distribution to kidney tissues and low nephrotoxicity was achieved. Since the Sta pattern recognition receptors (PRR) are overexpressed by macrophages infected with *Leishmania* parasites, Sta can be selected as a ligand targeting PRR and phosphatidylserine on the macrophage surface. Thereby, Sta presence on the nanoparticles surface may also be considered a target moiety [208]. The presence of TPGS emulsifier in the formulation, easily removed from NP surface, was able to increase AmB loading and EE, and to prompt an AmB response even against drug-resistant leishmanial strains.

Serrano et al. encapsulated AmB within amphiphilic *N*-palmitoyl-*N*-methyl-*N*,*N*-dimethyl-*N*,*N*,*N*-trimethyl-6-*O*-glycol chitosan (GCPQ) NPs that achieved 24.7% oral bioavailability [209]. The stable formulation resulted from electrostatic attractions between the quaternary ammonium groups of GCPQ and the carboxylate group of AmB combined with hydrophobic interactions between the polyene chain region of the drug and the palmitoyl chains of the GCPQ NPs. The AmB-GCPQ NPs showed comparable efficacy to L-AmB (AmBisome^®^) in candidiasis, aspergillosis, and VL disease models in vivo, demonstrating that oral NP uptake and organ targeting drives the activity of AmB-loaded GCPQ NPs. AmB-GCPQ target AmB to the liver, spleen, and lung (key organs of pathology in VL and systemic fungal infections) after oral administration, while sparing the kidney [209].

AmB-loaded chitosan-modified NLCs (AmB-CH-NLC) for fungal keratitis-targeted therapy showed successful in vivo penetration into the cornea with no observable irritation to the ocular mucosa of rabbits [210]. The AmB-CH-NLC exhibited improved bioavailability in the in vivo ocular pharmacokinetic study with potential to provide prolonged extraocular AmB delivery in fungal keratitis, a corneal infection of the eye mainly caused by *Candida* spp. and a leading cause of blindness resulting from corneal disease [210]. Previously, AmB-loaded lecithin/chitosan cationic NPs exhibiting pronounced mucoadhesive properties prepared by Chhonker et al. showed improved bioavailability upon topical ocular instillation in rabbits and increased precorneal residence time when compared to marked formulation Fungizone^®^ [244]. Biopolymers such as chitosan and alginate are known macrophage activators often used as immunomodulators to enhance immunological response in antimicrobial formulations.

Surface modification of SLN and NLC can be used to enhance drug retention at the absorption site. AmB-loaded NLCs coated with chitosan (ChiAmB NLC) were able to prevent expulsion of AmB upon exposure to simulated GI pH media, retaining up to 63.9% of the drug compared to 56.1% in the uncoated NPs [211]. The mode of AmB incorporation during formulation of the NLCs was crucial for the conformation exhibited by the drug [211]. The NLC formulations prepared by adding AmB in the undissolved state were more stable and promoted a safer monomeric form of the drug compared to NLC formulations containing dissolved AmB in the toxic (dimeric) aggregated state [211]. Furthermore, the coated formulation showed antifungal activity comparable to that of the free drug but reduced hemolysis and cytotoxicity [212]. The in vitro mucoadhesion properties of the chitosan coating were also observed ex vivo, resulting in higher retention time of the chitosan-coated formulation within the small intestine compared to the uncoated formulation [212].

NLC-loaded alginate hydrogels developed for oral delivery of AmB presented low cytotoxicity and high pH selectivity with no significant drug release in acidic buffer, suggesting their ability to protect AmB from gastric acid [245]. The NLCs were composed of solid lipid GSM, liquid lipid Mygliol 812N (a mixture of capric and caprylic acids), and surfactants Span 80, Kolliphor P188, and Tween 20, and were able to maintain their structure even after rehydration [245]. The drug release rate was found to be correlated with both alginate and cross-linking agent concentrations while the polymer swelling ratio was a determinant for drug delivery [245].

In the field of polysaccharide-based oil-core nanocapsules (NCs) and their eventual use in AmB encapsulation, Sombra et al. developed spherical NCs based on acetylated *Sterculia striata* polysaccharide (ASSP) [213]. This exudate polysaccharide is analogous to the commercial polysaccharide Karaya gum, and it has been already employed in the production of NPs through complexation with chitosan intended to deliver chloroquine drug [246]. Sombra et al. carried out a hydrophobic modification of SSP, employing its derivatives in the formation of stable NCs without the use of any surfactant [213]. The degree of substitution (DS) and the concentration of ASSP in the organic phase influenced particle size, polydispersity index and zeta-potential values, drug loading, and EE of NCs. AmB-NCs were found to reduce AmB aggregation, which is quite extraordinary considering the absence of a surfactant in their composition. Furthermore, loaded AmB was always in the monomeric state under the experimental conditions employed. AmB loaded NCs exhibited a sustained release of the drug up to 212 h, confirming its high potential as an AmB drug delivery platform. The strong interaction of the drug with the ASSP matrix hampers the exit of AmB to the medium, probably causing the slow release and the 99.2% of EE [213].

## 5. Current Amphotericin B Formulations in Clinical Trials

Several AmB formulations for antifungal or antiparasitic therapy are currently at different stages of clinical development (Table 3), including formulations for parenteral, oral, pulmonary, and topical skin delivery [247].

A phase IV RCT (NCT03814343) aiming to evaluate the effectiveness and safety of AmB in 30% DMSO solution in non-dermatophyte mold onychomycosis is currently recruiting [247]. The DMSO solvent can alter nail lipid concentration and keratin conformational structure, enhancing drug penetration into the intermediate nail plate [248]. In a pilot study (*n* = 8), non-dermatophyte mold onychomycosis treated with daily applications of topical AmB solution (2.0 mg/mL in a 1:1 mixture of DMSO and isopropyl alcohol) for 12 months resulted in clinical cure of all patients [248].

A recently completed phase II study to evaluate the safety and efficacy of a topical 3% AmB cream (Anfoleish^®^) for the treatment of uncomplicated CL in Colombia (NCT01845727) showed that the formulation was safe and well-tolerated, but efficacy results did not support the continuation of its clinical development for CL therapy [249]. The efficacy of antileishmanial creams can be hampered by epidermis thickening during lesion development that hinders drug absorption through the skin.

Topical pulmonary delivery with nebulized L-AmB (AmBisome^®^) alone (NCT02273661) or in combination with oral itraconazole (NCT03656081) is also being evaluated for allergic bronchopulmonary aspergillosis (ABPA) and chronic pulmonary aspergillosis therapy [247]. A retrospective study in India showed that intrathecal L-AmB (AmBisome^®^) administration for cryptococcal meningitis in HIV-infected patients (*n* = 18) was safe and well-tolerated [250]. The efficacy of intrathecal L-AmB in cryptococcal meningitis patients without AIDS has been evaluated in a recent phase IV clinical trial (NCT02686853) but results have not been posted yet [247]. The combination of intrathecal L-AmB (0.006 mg/kg/week) and oral voriconazole (30 mg/kg per dose twice daily) has shown a synergistic effect in the reduction of fungal load in a murine model of cryptococcal meningitis [251].

In an open-label, randomized trial of AmBisome^®^ alone or in combination with miltefosine to treat VL in HIV co-infected Ethiopian patients (NCT02011958), the combination regimen presented the highest documented efficacy and was recommended as first-line therapy for VL in East African HIV patients [252]. Short course combination regimens including AmBisome^®^, miltefosine, and paromycin for the treatment of VL have proved to be safe, well-tolerated, and not inferior in efficacy to AmBisome^®^ monotherapy in phase III clinical trials conducted in India (NCT00696969) and Bangladesh (NCT01122771) enrolling 634 and 601 patients, respectively, with no relapses or PKDL observed up to six months follow-up [253,254].

A steady-state global bioequivalence study of L-AmB for injection 50 mg/vial in VL patients under fed conditions (NCT03636659) has recently been completed and results have not been published yet [247]. A short-course AmBisome^®^ (15 mg/kg) regimen was shown to be safe and effective for PKDL therapy in Bangladesh (NCT03311607) and was recommended as a routine treatment option in the effort to eliminate VL in the Indian subcontinent [255]. A systematic review and meta-analysis of 31 prospective comparative clinical studies of AmB for the treatment of VL enrolling 6903 patients showed that in India (26 of the 31 studies) L-AmB was not inferior to AmB-DOC, which was as effective as miltefosine and apparently better than paromycin in achieving definitive cure [256].

Successful treatment of pulmonary blastomycosis with continuously infused AmB-DOC after failure of six-day therapy with L-AmB has been reported [257]. Continuous infusion of AmB-DOC (~1 mg/kg) over 24 h has been associated with fewer side effects compared with conventional rapid infusion over 4 h in a RCT enrolling 80 neutropenic patients with refractory fever and suspected or proven invasive fungal infections [258]. Clinical trials evaluating AmB-DOC in combination with flucytosine for HIV-associated cryptococcal meningitis (NCT04140461 and NCT04072640) are expected to start in 2020 [247].

Encochleated AmB (CAmB/MAT2203) developed by Matinas BioPharma is the only oral formulation of the drug currently in clinical trials. CAmB was shown to be safe and well-tolerated in women with moderate to severe vulvovaginal candidiasis in a recently completed phase II study (NCT02971007), with only seldom and mild GI side effects. However, clinical cure and mycological eradication rates in both the 200 mg and 400 mg arms did not reach standard of care fluconazole [259]. Other phase II studies for evaluation of safety and efficacy of CAmB (MAT2203) in the treatment of cryptococcal meningitis in HIV patients in Uganda (NCT04031833) and mucocutaneous candidiasis (esophageal, oropharyngeal, or vulvovaginal) refractory or intolerant to standard non-intravenous therapies (NCT02629419) are currently ongoing and results are expected by the end of 2021 [247].

## 6. Conclusions and Future Perspectives

The global burden of fungal infections that affect mostly developing countries and the emergence of antimicrobial resistance worldwide urges for effective and affordable AmB therapy. AmB is a membrane-acting broad-spectrum antibiotic in the clinic for more than half a century without emergence of significant antimicrobial resistance and remains the drug of choice in the treatment of systemic fungal infections and VL. However, the drug has severe side effects and its conventional formulation, AmB-DOC, is highly nephrotoxic. Lipid formulations with improved pharmacokinetics and safety profile have been developed but the approved marketed formulations are expensive and the recent introduction of generics as cheaper alternatives has raised the question of bioequivalence and safety. Furthermore, these formulations require i.v. administration and frequent dosing often associated with poor patient compliance. Therefore, development of alternative delivery systems for AmB that allow for more effective and noninvasive AmB delivery, such as lipid vehicles for oral, pulmonary or skin delivery, are urgent requirements. Nanotechnological strategies, such as solid lipid nanoparticles and nanostructured lipid carriers, provide promising approaches.

This review summarized novel lipid-based nanocarriers for AmB delivery that have been investigated over the last five years. Oral and topical AmB delivery has been accomplished in animal models of fungal infections and leishmaniasis, and also in clinical studies. Safety and efficacy of the most promising formulations for prophylaxis and treatment of antifungal and parasitic diseases remain to be established in future clinical trials.

## Figures and Tables

**Table 1:** Properties and clinical pharmacokinetic parameters of commercial amphotericin B parenteral formulations.

Formulation	Composition (Molar Ratio)	Structure	Size (nm)	Population (*n*)	Dose (mg/kg/day)	Duration	*C*_max_ (μg/mL)	AUC_0–24 h_ (μg/h/mL)	*t*_1/2_ (h)	CL (mL/h/kg)	*V*_d_ (L/kg)	Ref.
AmB-DOC (Fungizone^®^)	DOC:AmB (2:1)	Micellar dispersion	35	Mucocutaneous leishmaniasis (*n* = 5)	0.6 (i.v. 0.25 mg/kg/h), 42 days	Day 42	1.06 ± 0.14	17.06 ± 5.03	91.1 ± 40.9	29.2 ± 12.2 *^(a)^*	5.17 ± 2.6	[97]
				Neutropenic with fungal infection (*n* = 8)	1	Day 1	2.83 ± 1.17	28.98 ± 15.46	15.23 ± 5.25	33.01 ± 14.33	0.56 ± 0.15	[98]
AmB-DOC in Intralipid^®^ 20% (admixture)	Soybean oil 20% *w*/*v*, egg yolk PLs 1.2% *w*/*v*, glycerin 2.25% *w*/*v*, AmB 1 mg/mL	Fat emulsion	<1000	Neutropenic with fungal infection (*n* = 8)	1	Day 1	1.46 ± 0.61	17.22 ± 11.15	11.44 ± 5.18	62.97 ± 35.51	1.04 ± 0.51	[98]
ABLC (Abelcet^®^)	DMPC:DMPG (7:3)-AmB (1:1)	Ribbons	1600–11,000	Mucocutaneous leishmaniasis (*n* = 8)	5 (2 h-inf.), 5 days	Day 5	1.70 ± 0.83	9.50 ± 1.36	173.4 ± 78.0	408.2 ± 61.9 *^(a)^*	131.0 ± 57.7	[97]
				Fungal infections (*n* = 17)	5 (2-h inf.), 10–17 days	Last day	2.39 ± 1.58	19.17 ± 4.43	393 ± 486	270 ± 70	147 ± 144	[99]
ABCD (Amphotec^®^, Amphocil^®^)	Cholesteryl sulfate:AmB (1:1)	Disks	(122 ± 48) × 4.3	Critically ill patients (*n* = 5)	4 (4 h-inf.), 5 days	Single dose	0.65 ± 0.26	4.24 ± 1.14	28.54 ± 18.97	320 ± 230	8.89 ± 2.64	[100]
						Day 5	1.24 ± 0.53	14.22 ± 7.54	19.56 ± 8.44	190 ± 130	4.65 ± 2.01	[100]
				Bone marrow transplant recipients (*n* = 51)	4 (4 h-inf.), 10 days	Steady state *^(b)^*	2.8	42	29.8	112	4.08	[101]
L-AmB (AmBisome^®^)	HSPC:DSPG:cholesterol:AmB (2:0.8:1:0.4)	SUV	60–80	Critically ill patients (*n* = 16)	3.0 (1 h-inf.)	Steady state	14.4 (6.4–89.0)	171 (53.1–1380)	13.05 (8.70–41.40)	0.311 (0.031–0.807) *^(a)^*	0.421 (0.055–0.932)	[102]
				Neutropenic with fungal infection (*n* = 12)	5.0 (1 h-inf.), 8 days	Day 1	57.6 ± 21.0	269 ± 96	6.4 ± 2.1	21 ± 14	0.22 ± 0.17	[103]
						Last day	83.0 ± 35.2	555 ± 311	6.8 ± 2.1	11 ± 6	0.11 ± 0.08	[103]
				Immuno-compromised patients with IFI (*n* = 8)	7.5 (2 h-inf.), 23 days	Day 1	75.9 ± 58.4	692 ± 834	6.8 ± 1.9	23 ± 14	0.22 ± 0.18	[104]
						Last day	144.3 ± 61.6	1286 ± 973	6.5 ± 3.4	11 ± 13	0.08 ± 0.08	[104]
Indian L-AmB (Fungisome^TM^)	SPC:cholesterol (7:3)-AmB (1:45) 2.2% *w*/*w* in normal saline	MLV	2743–3454	Systemic fungal infections (*n* = 12)	1 (1 h-inf.), 3 days	Last day	1.01 ± 0.06	11.43 ± 0.91	17.2 ± 1.8	91.7 ± 8.9	2.28 ± 0.30	[105]

ABCD, amphotericin B colloidal dispersion; ABLC, amphotericin B lipid complex; AmB, amphotericin B; AmB-DOC amphotericin B deoxycholate; AUC, area under the concentration–time curve; CL, total body clearance; *C*_max_, peak plasma concentration; DMPC, dimyristoyl phosphatidylcholine; DMPG, dimyristoyl phosphatidylglycerol; DOC, deoxycholate; DSPG, distearoyl phosphatidylglycerol; HSPC, hydrogenated soy phosphatidylcholine; IFI, invasive fungal infection; inf., infusion; i.v., intravenous; L-AMB, liposomal amphotericin B; MLV, multilamellar vesicles; *n*, number of subjects; PL, phospholipid; SPC, soy phosphatidylcholine; SUV, small unilamellar vesicles; *t*_1/2_, half-life; *V*_d_, apparent volume of distribution; *w*/*w*, weight/weight; *^(a)^*assuming mean body weight of 70.0 kg; *^(b)^* Predicted values obtained from population modeling. Data presented as mean ± standard deviation or mean (range).

**Table 2:** Lipid-based systems for delivery of amphotericin B currently under development.

Delivery System	Adm. Route	Size, nm (PI)	EE, %	Formulation Composition and Preparation Method	Main Outcomes and Limitations	Ref.
Oleic acid (OA) conjugate (amide prodrug)	Oral	N/A	N/A	AmB-OA conjugate. Synthesis via CDI chemistry.	Conjugation to OA improved AmB stability at gastric pH and permeability in Caco-2 monolayer model with cell viability >90% and reversible TEER reduction. Metabolism of AmB-OA into AmB > 80% in liver homogenate. AmB-OA showed differential aggregation behavior with no evidence of hemolytic or kidney (HEK 293 cells) toxicity in vitro. Oral AmB-OA given to rats (10 mg/kg in PBS as gavage vehicle) significantly increased *C*_max_ and AUC compared to AmB i.v. and AmB-OA admixture. Histopathological studies did not show kidney or liver damage in animals treated with oral AmB-OA conjugate.	[161,162]
Molecular umbrella conjugate (amide prodrug)	N/A	N/A	N/A	AmB-di-walled molecular umbrella (cholic acid walls and spermidine scaffold) conjugate. Synthesis via CDI chemistry.	Conjugation reduced hemolytic activity and in vitro cytotoxicity to kidney (HEK 293) cells. The conjugate retained in vitro antifungal efficacy against *C. albicans*, *Candida glabrata*, *Cryptococcus neoformans,* and *Cryptococcus gatti* with MIC and MFC values in the range of 1–2 μM and 2–4 μM, respectively.	[163]
SDCS micelles	Pulmonary	73 ± 0.9		AmB:SDCS (1:2 molar ratio). Lyophilized dry powder.	Jet nebulization of anionic SDCS micelles (ζ –40 mV) produced aerosols suitable for inhaler use (MMAD 1.0 μm, FPF 81.4%, and GSD 2.1). Enhanced in vitro antifungal activity against *C. albicans*, *C. neoformans,* and *Saccharomyces cerevisiae*, antileishmanial against *Leishmania tropica* promastigotes (IC_50_ 0.021 μM). Not cytotoxic in vitro (MTT assay) to kidney (HK-2, 293T/17), bronchial epithelial (HBE1), lung cancer (A549, Calu-3), and macrophage (NR8383, RAW 267.4) cell lines at conc. up to 8 μg/mL (cell viability > 90%). In vitro phagocytosis by alveolar macrophages (NR8383). Not nephrotoxic in vivo after 7 days of regular dosing (1.5 mg/kg/day) to rats by intratracheal instillation. Higher drug conc. in lung (7.5 μg/g) and lower in kidney (0.06 μg/g).	[124,164,165,166]
LAA micelles	Oral *^(a)^*	N/A	63.4	AmB-loaded LAA micelles. Self-assembly in PBS (pH 7.4) at r.t.	LAA micelles (CMC 0.612 mM) deaggregated AmB in a concentration-dependent manner with a more pronounced effect at higher LAA conc. In vitro activity against *C. albicans* (ATCC 10231), MIC 3.12 μg/mL.	[167]
LAA-BS mixed micelles	Oral, topical *^(a)^*	N/A	49–61	LAA-BS mixed micelles (1:1 molar ratio) loaded with AmB. Self-assembly in PBS (pH 7.4) at r.t.	The presence of LAA with dimeric structure in LAA-NaC (CMC 1.05 mM) and LAA-NaDC (CMC 1.74 mM) improved AmB solubilization compared to pure BS micelles. In vitro activity against *C. albicans* (ATCC 10231), MIC (μg/mL) 2.50 (LAA-NaC) and 6.25 (LAA-NaDC). UV spectroscopy showed monomeric AmB in LAA-BS mixed micelles at 10 mM. LAA-NaDC formed shear-shinning gels at higher NaDC conc. (>10 mM) that can provide interesting topical DDS.	[168]
Surface-modified liposomes (SML)	Parenteral	204.4 ± 0.34 (0.22)	95	Unsaturated SPC:saturated SPC:cholesterol (1:1:1 molar ratio) with modified ligand (3% *w*/*w*) and AmB (105 μg/mg lipid). Thin film hydration method.	Alginate hydrophobized by conjugation with Sta produced anionic SML (ζ –19.21 mV) with enhanced cellular uptake in macrophage (RAW 264.7) cells attributed to receptor-mediated endocytosis. In healthy mice, SML (5 mg/kg single i.v. dose) resulted in improved PK profile, higher accumulation in liver and spleen (with no histopathological damage to the organs), and lower accumulation in kidney compared to conventional (unmodified) liposomes and AmB solution. SML displayed enhanced anti-leishmanial activity against both promastigotes (IC_50_ 39.7 nM) and amastigotes (IC_50_ 29.1 nM) of *Leishmania donovani* (Dd8) compared to conventional liposomes and AmB solution.	[169]
Magnetic liposomes (MLP)	Intracarotid	240 ± 11 (0.092)	79.32	SPC:cholesterol (5:1 weight ratio) and AmB:Fe_3_O_4_ (1:2 weight ratio). Film dispersion-ultrasonication method.	Good magnetic responsiveness (saturation magnetization 32.54 memu/g at r.t.). AmB-MLP crossed the BBB enhancing AmB conc. in the brain under an applied magnetic field after carotid artery injection to rats (1 mg/kg). AmB-MLP may be used for simultaneous MRI and AmB delivery in brain fungal infections.	[170]
Stigmasterol-based liposomes (DSHemsPC)	Parenteral	111.6 ± 1.0 (0.21)		DSHemsPC:DMPC:DMPG:AmB (1.25:5:1.5:1 molar ratio). Thin film hydration method.	Anionic liposomes (ζ –25.3 mV) were less hemolytic (IC_50_ 42.6 mg/mL) than AmBisome^®^ (IC_50_ 11.6 mg/mL). In vitro antifungal activity (MIC 0.07–2.3 μg/mL) against *C. albicans* (PTCC 5027), *C. glabrata* (PTCC 5297), *A. fumigatus* (PTCC 5009), *A. terreus* (PTCC 5021), and *A. flavus* (PTCC 5006), anti-leishmanial against *Leishmania major* promastigotes (ED_50_ 1.4 μg/mL) and amastigotes (ED_50_ 0.14 μg/mL). Biodistribution studies in mice showed higher AmB conc. in liver and spleen and lower in kidney. In mouse models of both acute and established lesions (*L. major* MRHO/IR/75/ER), multiple doses (5 mg/kg i.v.) of AmB-DSHemsPC cleared parasites from liver and spleen but less effectively from footpad, suggesting more suitability for VL treatment than CL.	[171,172]
Transfersomes, UDL	Skin	106 ± 6 (0.14)	75	SPC:Tween 80:AmB (86:43:0.1 weight ratio). Thin film hydration method.	AmB-UDL encapsulated AmB in its monomeric form. In vitro antifungal activity (MIC 0.06–0.25 μg/mL) against ATCC *Candida* strains (*C. albicans* 10231, *C. tropicalis* 750, *C. glabrata* 90030, *C. krusei* 6258, *C. parapsilosis* 22019) and clinical isolates of *C. albicans* at conc. not toxic to human keratinocyte (HaCaT) and murine monocyte/macrophage (J774) cells. AmB-UDL at 1.25 μg/mL exhibited 100% and 75% activity against promastigote and amastigote forms of *L. braziliensis*, respectively. Non-occlusive incubation (1 h) of human skin explants with AmB-UDL revealed drug penetration into deep epithelial layers and much higher drug accumulation in skin (1.8 ± 0.1 μg/cm^2^) compared to AmBisome^®^ (0.045 ± 0.002 μg/cm^2^).	[173]
Nanoethosomes	Skin	258 ± 2 (0.261)	89.1	SPC:ethanol:AmB (3:30:0.1 weight ratio) in Carbopol^®^ gel (1.5% *w*/*w*).	Nanoethosomes containing 30% (*w*/*w*) ethanol showed enhanced skin permeability compared to a marketed gel formulation (AmB 0.1% *w*/*w*). Enhanced in vitro activity against *C. albicans*. No skin irritation observed in vivo (Draize test).	[174]
Ergosterol-based liposomes (Kalsome^TM^10)	Parenteral	119.5 ± 14.85		PC:ergosterol:AmB (5:2:1.8 molar ratio) in 0.9% saline. Thin film hydration method.	Mixed lamellarity liposomes, require sonication before administration to reduce particle size. Kalsome^TM^10-mediated anti-leishmanial effect is dependent on endocytosis by host macrophages. Kalsome^TM^10 induced apoptosis in both promastigote and intracellular amastigote forms of *L. donovani* but not on mammalian host macrophages. Mechanistic studies showed increased ROS production, caspase-like activity, and DNA fragmentation in Kalsome^TM^10-treated promastigotes and amastigotes. In *L. donovani*-infected mice, Kalsome^TM^10 to (7.5 mg/kg triple i.v. dose) suppressed parasite burden, shifting immune response from Th2 (IL-10 and TGFβ production) to Th1-type (IL-12 and IFNγ production).	[175,176,177]
PEGylated cationic liposomes	Vaginal	400–500	50–60	DOPE:DOTAP:cholesterol (4:5:1 molar ratio) and 6 mol% DSPE-PEG2k in thermosensitive poloxamer gel composed of P407:P188 (15:15 *v*/*v*).	PEGylation enhanced AmB solubility and encapsulation efficiency. The formulation showed a sol-to-gel transition at body temperature of 37 °C. PEGylated cationic liposomes (ζ 40–60 mV) were more stable and less toxic to kidney (HEK 293) cells than the free drug.	[178]
Sophorolipid (SL) niosomes	N/A	80	63.20	Acidic SL:cholesterol:DCP (10:0.9:0.64 molar ratio) and AmB in 0.9% saline. Thin film hydration method.	Affordable SLs produced by *S. bombicola* from renewable low-cost substrates (rice bran and cottonseed oil). Higher *C. albicans* (clinical strain SC5314) anti-biofilm effect of AmB-SL niosomes after 24 h incubation (BEC_50_ 0.195 μg/mL) compared to AmB alone (BEC_50_ 0.390 μg/mL). Absence of pseudohyphae on mature biofilms treated with niosomal AmB but not with marketed liposomal AmB (Phosome^®^).	[179]
Cholesterol-based NEs	Parenteral *^(a)^*	169 ± 0 (0.11 ± 0.02)	99 ± 1	5% MCT, 1.5% polysorbate 80, 0.5% cholesterol, 0.2% Sta, 0.01% α-tocopherol, 2.25% glycerol, AmB (1.99 ± 0.01 mg/mL); pH 7.44. Hot homogenization method.	Cytotoxicity studies on J774 (ATCC/TIB-67TM) murine macrophages (24 h exposure) CC_50_ 1.8 ± 0.8 μg/mL. In vitro efficacy against intracellular amastigotes of *L. amazonensis* (IFLA/BR/67/PH8), IC_50_ 0.11 ± 0.03 μg/mL. Stability (ζ 53 ± 2 mV) for at least 180 days. Increase of Sta conc. enhanced cytotoxicity and antifungal activity. NEs less toxic than conventional AmB (reduced of self-associated AmB in lipid nanocarrier). Selectivity index was significantly higher than that of conventional AmB.	[180]
Sefsol 218-based NEs	Skin	67.32 ± 0.8 (0.23)		Sefsol-218 (12.5% *w*/*w*), Tween 80:PEG400 S_mix_ 2:1 (33.45% *w*/*w*) AmB; pH 7.4. Slow spontaneous titration method.	Cumulative amount of AmB permeated (24 h): 425.36 ± 1.9 μg. Permeation flux rate 17.8 ± 0.5 μg/cm^2^/h (higher than AmB solution and Fungisome^®^). AmB deposition in abdominal albino rat skin, after first 6 h: 35.6 ± 2.0%. AmB undecomposed in NE (ζ –37.3 mV), after 90 days of storage: 99.3% (5 °C), 96.7% (25 °C), 87.1% (40 °C). Minimal aggregation behavior at pH 6.8 and pH 7.4.	[181]
Capmul-based NEs	Skin	49.5 ± 1.5 (0.33)		Capmul PG8 (15% *w*/*w*), LAB-PEG400 S_mix_ 1:2 (24% *w*/*w*), AmB; pH 7.4. Slow spontaneous titration method.	ZOI: 19.1 ± 1.4 mm (*Aspergillus niger*, MTCC 282), 22.8 ± 2.0 mm (*C. albicans*, MTCC 4748). AmB-NE (ζ –24.59 mV) in vitro release (slow and sustained release): 46.1 ± 3.7% (60 min). Ex vivo skin permeation flux rate: 22.88 ± 1.7 μg/cm^2^/h. AmB deposited in abdominal albino rat skin, after first 6 h: 74 ± 5.6%.	[182]
NEs with antifungal excipients	Skin	74.8 ± 4.1 (0.21)		Peceol (19.4% *w*/*w*), LAB:PG S_mix_ 1:3 (14.9% *w*/*w*), AmB; pH 6.8. Slow spontaneous titration method.	ZOI: 21.8 ± 1.5 mm (*A. fumigatus*), 19.7 ± 1.2 mm (*C. albicans*).AmB-NE (ζ –33.2 mV) in vitro sustained release: 19.8 ± 1.1% (90 min). Enhanced ex vivo rat skin permeation-deposition (skin permeation flux rate: 21.62 ± 1.6 μg/cm^2^/h; AmB deposition: 84.7 ± 9.3 μg).	[183]
Castor oil-based NEs	Skin	128.40 ± 12.71 (0.27 ± 0.05)	95 ± 2	5% castor oil, 55% LAB:Plurol^®^ oleique (5:1), 40% Transcutol^®^ P, 0.50% (*w*/*w*) AmB; pH 7.42 ± 0.53. Aqueous titration method.	Newtonian behavior, viscosity 12.20 ± 0.28 mPa·s. In vitro sustained release (without burst effect): 100% of AmB after 75 h. AmB retention 17.76 μg/g/cm^2^ after 36 h of skin application. Antifungal activity against *C. albicans* (ATCC 10231), *C. glabrata* (ATCC 66032), *C. parapsilosis* (ATCC 22019), *A. brasiliensis* (ATCC 16404), with MICs of 0.78, 0.39, 0.19, 0.13, respectively. Ex vivo permeation studies on women skin suggests no theoretical systemic absorption: all participants exhibited TEWL values in the normal range (except after 2 h, possibly caused by the effect of Transcutol^®^ P on skin).	[184]
SEDDS (iCo-010)	Oral	≈200		60/40 (*v*/*v*) mono- and diglycerides (Peceol/Gelucire 44/14, lauroyl macrogol-32-glycerides) with vitamin E-TPGS. Mixing, mild heating and stirring (45 °C for 1–2 h).	Stability: >75% (over 60 days, 30 °C and 43 °C); >95% (4 h in SIF). In vivo antileishmanial activity in murine model of VL (BALB/c mice infected with *L. donovani* promastigotes): <99% inhibition (10 mg/kg orally, twice daily, 5 days); 95% inhibition (20 mg/kg orally, once daily, 5 days). Efficacy in a mouse model of systemic candidiasis (*C. albicans* ATCC 18804): 69–96% reduction of fungal burden in mouse tissues with oral iCo-010 at 5, 10, and 20 mg/kg daily for 5 days, when compared to untreated animals.	[185,186,187,188]
SNEDDS	Topical, oral *^(a)^*	FA: 27.70 ± 0.5 (0.187); FB: 30.17 ± 0.7 (0.171)		Formulation A (FA): 35% DMSO, 20% Captex 300, 45% Cremophor EL; Formulation B (FB): 35% DMSO, 20% Captex 300, 35% Cremophor RH, 10% Tween 80. Vortex mixing and sonication.	Diffusion ability on porcine GIT mucus in up to 8 mm of mucus: FA, 1.45%; FB, 1.37%. In vitro permeation across Caco-2 cells monolayer (0.5% AmB-loaded SNEDDS): FA, 10%; FB, 11%. In vitro evaluation of cytotoxicity (Caco-2 cell lines): FA, 89%; FB, 86.9%. Spreading efficiency: on 12 cm^2^ of buccal mucosa, FA, 45 min; FB, 50 min; on 14 cm^2^ of ulcerated skin model, FA, 55 min; FB, 60 min. In vitro anti-leishmanial activity against *L. tropica*, IC_50_: FA, 0.017 ± 0.005 μg/mL (promastigote), 0.025 ± 0.003 μg/mL (amastigote); FB, 0.031 ± 0.006 μg/mL (promastigote), 0.056 ± 0.004 μg/mL (amastigote). At 0.1 μg/ml concentration, FA and FB formulations successfully released AmB in infected macrophages and killed 100% of *Leishmania* parasites.	[189]
PHY cubosomes	Oral	206.31 ± 4.66 (0.12)	91	PHY, Pluronic F127 (20% *w*/*w*), AmB (7% *w*/*w*). Hydrotrope (methanol) dilution method.	Stable in SGF and SIF, enhanced uptake by Caco-2 cells without cytotoxicity to the Caco-2 monolayer. In vitro sustained AmB release (PBS pH 7.4, 37 °C), with slower release from PHY than from GMO cubosomes (80% vs. 90% release after 120 h). Oral administration to rats (10 mg/kg) enhanced oral bioavailability (increased *C*_max_ and AUC) in the order AmB-PHY > AmB-GMO >> AmB due to PHY higher acid resistance.	[190]
GMO cubosomes	Oral	192.3 ± 10.8 (0.20)	94	GMO:poloxamer P407 (18:1 weight ratio), AmB. O/W emulsion technology.	Stable in SGF and SIF, enhanced uptake by Caco-2 cells via clathrin- and caveolae-mediated endocytosis. Plasma drug profile showed a sustained release of AmB over 5 days with no differences found in serum creatinine and BUN levels prior to and 24 h after oral administration of AmB-GMO (10 and 20 mg/kg single dose) to rats. In a rat model of systemic candidiasis (*C. albicans* ATCC 18804), oral administration of AmB-GMO (1, 5, and 10 mg/kg thrice daily for 5 days) reduced fungal burden only in kidneys, consistent with dose-dependent response in kidney tissue.	[191,192]
GMO cubic phase gel	Intra-articular	N/A	N/A	GMO 55%, soybean oil 5%, hyaluronic acid 0.75%, water 15%, ethanol 10%, PG 15%, AmB 0.1% *w*/*w*. Dispersion method.	The hydrolipid formulation showed good syringeability forming a shear-shinning gel. In vitro drug release profile (PBS, pH 7.4) at 37 °C showed long-term sustained release lasting several weeks. Intra-articular administration to rabbits corroborated long-term sustained release of AmB with no signs of inflammation at the injected joint.	[193]
Cochleates (CAmB)	Oral	407.3 ± 233.8		Soy lecithin (enriched with 50% *w*/*w* PS-CaCl_2_): AmB (1:10 molar ratio). Aqueous-aqueous hydrogel binary system.	Oral CAmB + flucytosine more effective than oral fluconazole against *C. neoformans* H99 (ATCC 208821) in a murine model of cryptococcal meningoencephalitis without toxic side effects. Fluorescence imaging demonstrated brain transport and accumulation of CAmB. Oral CAmB (25 mg/kg/day) and AmB-DOC (5 mg/kg/day i.p.) plus oral flucytosine (250 mg/kg/day) for 3 weeks had similar efficacy and immunological profile in treated mice.	[194,195]
Cochleates modified with adjuvant Finlay (AFCo3)	Intra-peritoneal	5000–10,000		Detoxified LPS from *Neissera meningitides* B (87% *w*/*w*), CaCl_2_, AmB. Resuspension-sedimentation method.	In vitro activity against *Sporothrix schenckii* (ATCC 16345) with MIC 0.25 μg/mL and MFC 0.5 μg/mL. Enhanced intracellular fungicidal activity in peritoneal macrophages with stimulation of TNF-α, IL-1β, and NO release in vitro and ex vivo in mouse splenocytes. Reduced spleen and liver fungal burden after 5-day treatment (5 mg/kg/day i.p.) in a mouse model of systemic sporotrichosis and induced Th1/Th17 response with no significant changes in BUN and serum creatinine levels.	[196]
Nanodisk (ND)	Sinonasal *^(a)^*	44–80		DMPC:DMPG (7:3 weight ratio), ApoA-I, AmB. Thin film method; sonication and dialysis after addition of AmB and ApoA-I.	HSNE exposed to toxic conc. of AmB-ND (18 h): apical membranes permeable to K^+^ ions at 10 μg/mL AmB; reduction of apical cell K^+^ permeability at 75 μg/mL AmB with 85% reduction of LDH and no increase in LDH release at 150 μg/mL AmB. In vitro expression of *A. fumigatus* (ATCC 13073) conidia after 4 h exposure to AmB-ND (10 μg/mL) smaller than exposure to AmB; AmB-ND (50 μg/mL) RNA expression without statistical significance between AmB and AmB-ND. AmB-ND protected human nasal epithelia membranes from AmB toxicity.	[197,198]
SLN gel	Skin	111.1 ± 2.2 (0.13 ± 0.04)	93.8 ± 1.8	AmB:lipid (1:10 weight ratio), Pluronic F127 (0.25% *w*/*v*). Solvent diffusion method in aqueous system.	Antifungal activity against *Trichophyton rubrum* (ATCC 28188, KWIK-STIK 0444P), ZOI (72 h) 2.81 ± 0.13 mm. Stability (ζ –23.98 ± 1.36 mV) at 2–8 °C and 25 ± 2 °C, for 3 months. AmB in aqueous phase: 90.2 ± 1.1% (compritol ATO 888), 96.5 ± 1.4% (Precirol ATO 5, selected for preparation of SLNs gel), and 72.1 ± 2.7% (stearic acid). Ex vivo permeation studies on abdomen skin of female albino Wistar rats: AmB efflux 22.34 μg/cm^2^. PII (SLN gel) 0.11 ± 0.19 Higher skin deposition, lower skin irritation, high antifungal activity, localized delivery with minimal side effects.	[199]
Glyceride dilaurate-based SLN (AmbiOnp)	Oral	392.8 ± 6.97		GDL, PC-enriched lecithin, PEG-660-12-hydroxystearate, AmB. Probe sonication-assisted nanoprecipitation technique.	Easy redispersion of AmbiOnp in water (3 months, 2–8 °C). Significant increase in particle size at 25 and 40 °C after 3 months. AmB content in AmbiOnp (ζ –27.9 ± 0.2 mV) ≈ 100% (after 1 month), showing significant reduction after 3 months when stored at 25 °C and 40 °C. In vitro antifungal activity against *C. albicans* in SGF, MIC 7.812 μg/mL.In vivo PK studies (adult female Sprague-Dawley rats): C_máx_ 1109.31 ± 104.79 ng/mL, AmbiOnp (3.6 mg/kg of AmB) oral, 24 h, comparable to C_máx_ 1417.49 ± 85.52 ng/mL Fungizone^®^ (0.8 mg/kg of AmB), i.v. Low renal tissue levels of AmB in AmbiOnp at 8 h: 84.50 ± 22.896 ng/mL; without detectable levels post 8 h.	[200,201]
Sesame oil-based NLCs	N/A	AmB-NLC: 79.5 ± 2.7 (0.26 ± 0.01); LYO-AmB-NLC: 179.7 ± 2.1 (0.23 ± 0.00)	AmB-NLC: 98.1 ± 1.3; LYO-AmB-NLC: 96.9 ± 2.1	GMS:sesame oil (7:3 weight ratio), Pluronic F68 (3% *w*/*w*), AmB. Ultrasonic cavitation homogenization method.	AmB-NLC (ζ –14.8 ± 1.9 mV): LC 0.196 ± 0.003; LYO-AmB-NLC (ζ –14.6 ± 2.6 mV): LC 0.194 ± 0.004. AmB-NLC in vitro release profile (24 h after particle preparation), fitted the Baker–Lonsdale model; AmB controlled release for 72 h.	[202]
Sta-OA NLCs	Pulmonary	659.7 ± 1.20 (0.27 ± 0.12)	77.10 ± 5.50	Sta:OA (6:4 weight ratio), AmB. Solvent diffusion method followed by AmB loading and spray-drying.	In vitro sustained drug release (88.2% up to 40 h) and reduced dose dumping. Better in vitro antifungal activity over control against *A. fumigatus* (MTCC 5186). PK, histopathology, hematological, and in vivo biodistribution studies demonstrated localized AmB delivery for prolonged period after pulmonary administration to rats and reduced nephrotoxicity.	[203]
PEG-DSPE micelles	Parenteral	23.2 nm		AmB-DOC:PEG-DSPE micelles (1:20, 1:40, and 1:90 molar ratio) in 0.9% saline.	Reconstitution of Fungizone^®^ with PEG-DSPE micelles in saline facilitated co-delivery of monomeric AmB and sodium supplementation. More extensive AmB deaggregation achieved with AmB-DOC:PEG-DSPE at 1:90 molar ratio, decreasing the size of AmB-DOC aggregates from 3310 nm (in saline) to 23.2 nm in mixed DOC-PEG-DSPE micelles. Reduced in vitro hemolytic activity (bovine RBCs) and in vivo renal toxicity in rats after 1.5-h infusion (2 mg/kg/day) for 3 days compared to conventional formulation. Similar in vitro fungicidal activity against *S. cerevisiae* (ATCC 9763) and *C. albicans* (K1 strain). Simple preparation from commercially available AmB-DOC and FDA-approved PEG-DSPE excipients.	[204]
Lecithin-based polymeric hybrid micelles (Ambicelles)	Oral, parenteral	187.20 ± 10.15 (0.51)	90.14	AmB:lecithin:DSPE-PEG2k (1:1:10 weight ratio). Thin film hydration method.	Ambicelles improved AmB solubility from 0.001 to 5 mg/mL. Increased bioavailability in rats compared to Fungizone^®^ after single i.v. (0.8 mg/kg) and oral (10 mg/kg) doses. Reduced in vitro cytotoxicity against human colon adenocarcinoma (HT29) cells. Organic solvent (methanol:dichloromethane 19:1) required to dissolve AmB, lecithin and polymer.	[205]
MPEG-CS-LNA hybrid micelles	Parenteral	257.94 ± 10.42 (0.181 ± 0.028)	82.3	AmB:MPEG-CS-LNA (1:7 weight ratio). Dialysis method.	Cationic hybrid micelles (ζ 17.53 ± 0.15 mV) with low CMC (0.2138 mg/mL) enhanced drug solubilization up to 1.64 mg/mL. Reduced hemolytic effect in vitro and decreased nephrotoxicity in vivo compared to i.v. AmB, with improved PK profile. Enhanced in vivo fungal cellular uptake due to combined inducement of LNA and CS.	[206]
Hybrid NE with lipo-polymeric nanoparticles	Topical *^(a)^*	50 ± 10		Tween 80: canola oil (1.18 weight ratio), Carbopol^®^ (1% *w*/*w*), HPMC (0.03% *w*/*w*). Solvent evaporation-emulsion technique.	In vitro antifungal activity against *A. fumigatus* (FCBP 66), *Aspergillus flavus* (FCBP 0064), *A. niger* (FCBP 0198), *Fusarium solani* (FCBP 0291): ZOI 14 mm, 8 mm, 10 mm, 19,5 mm, respectively. In vitro anti-leishmanial activity (*L. tropica* promastigotes): 50% killing rate at 0.2 μg/mL AmB; 100% mortality at 20 μg/mL AmB. LC_50_ against *L. tropica*: 0.743 μg/m (AmB emulsion in water); 0.190 μg/m (AmB emulsion in DMSO). Physical stability for more than three months; AmB proved to be undamaged in the nanoformulation.	[207]
Cationic Sta lipid–polymer hybrid NPs (LPNPs)	N/A	198.3 ± 3.52 (0.135 ± 0.03)	96.1 ± 2.01	PLGA, AmB (10% *w*/*w*), Sta (1% *w*/*v*), TPGS. Modified W/O/W double emulsification method.	Cationic hybrid NPs (ζ 31.6 ± 1.91 mV) provided sustained AmB release. Antileishmanial activity against *L. donovani* (MHOM/IN/80/Dd8): in vitro (J774A.1 intra-macrophage amastigotes) IC_50_ 0.16 ± 0.04 μg AmB/mL); in vivo (Syrian golden male hamsters), 89.41 ± 3.58% parasite inhibition against VL models. Antileishmanial activity enhancement due to macrophage targeting potential, Th-1 biased immune-alteration mediated by drug-free LPNPs and synergistic activity of Sta lipid component. Reduced level of nephrotoxicity markers and minimal distribution to kidney tissues.	[208]
Palmitoyl-modified chitosan NPs (GCPQ)	Oral	35 and 216	N/A	GCPQ (16.9 mol% palmitoylated, 16.5 mol% quaternized, HI 1.02; 5 mg/mL), AmB (1 mg/mL). Polyelectrolyte complex formation.	The bimodal size formulation is due to an equilibrium between AmB loaded NPs and empty micelles formed by self-assembly of amphiphilic GCPQ. AmB-GCPQ NPs delivered the drug specifically to liver, lung, and spleen while sparing the kidney. Mucoadhesive GCPQ NPs were taken up by the gut enterocytes with bioavailability of 24.7%. AmB-GCPQ NPs (5 mg/kg/day orally for 10 days) cleared spleen and liver fungal load in a systemic murine model of candidiasis and were as effective as parenteral AmBisome^®^ in a murine model of VL. Oral AmB-GCPQ NPs (7.5 or 15 mg/kg/day for 7 days) reduced fungal burden in a murine model of disseminated aspergillosis and was statistically similar to AmBisome^®^ (5 mg/kg/day i.v.).	[209]
Chitosan-modified NLCs (CH-NLC)	Ocular	185.4 ± 5.9 (0.20 ± 0.08)	90.9 ± 3.9	AmB-chitosan NLC. Emulsion evaporation-solidification method.	Cationic chitosan-modified NLC (ζ 27.1 ± 2.9 mV) provided in vitro sustained drug release. Improved bioavailability of AmB-CH-NLC suggested by in vivo ocular PK study (*t*_1/2_ 2.37 h vs. 1.24 h and 0.16 h for uncoated formulation and AmB eye drops, respectively). In vivo corneal penetration study showed successful penetration into the cornea with no obvious irritation to the ocular mucosa of rabbits’ eyes. AmB-CH-NLC may be a promising system for ocular delivery of AmB in fungal keratitis-targeted therapy.	[210]
Chitosan-coated NLCs	Oral *^(a)^*	394.4 ± 6.4 (0.44 ± 0.03)	86.0 ± 3.0	Beeswax, coconut oil, Tween 80, lecithin, AmB. Homogenization-ultrasonication technique and further coating with chitosan (1:40 *v*/*v*).	In vitro slow release profile (26.1% drug release in 5 h). Prevention of AmB expulsion upon exposure to simulated GI pH media (63.9% drug retention vs. 56.1% in the uncoated formulation). Chitosan-coated NLCs showed mucoadhesive properties in vitro and ex vivo on excised rat intestinal tissue; higher retention time within the small intestine (84.2 ± 5.1% adhesion vs. 55.8 ± 16.1% with the uncoated formulation). Coated formulation was less cytotoxic than the free drug to RBCs and HT-29 cells, but with comparable in vitro antifungal activity against *C. albicans* (ATCC 90028).	[211,212]
Acetylated *Sterculia striata* polysaccharide (ASSP) NCs(DS 1.68 and DS 1.35)	N/A	NC1.68: 274.1 ± 8.6 (0.181); NC1.35: 277.3 ± 9.5 (0.149)	NC1.68: 99.2 ± 1.3; NC1.35: 80.5 ± 1.6	ASSP (1.0 mg/mL), Miglyol812^®^, AmB (0.25 mg/mL). Spontaneous emulsification.	NC1.68 (ASSP concentration, 0.5 mg/mL) and NC1.35 (ASSP concentration, 1.0 mg/mL) loaded AmB in a monomeric form. AmB-NC1.68: MIC 0.25 μg/mL against 5 strains of *C. albicans* (ATCC 90028, LABMIC 0104, LABMIC 0105, LABMIC 0106). In vitro controlled release of AmB: 3.6 ± 0.5% after 1 h; 49.1 ± 0.4% after 72 h; complete release after 212 h. NC1.68 showed potential to be employed as AmB DDS.	[213]

*^(a)^* intended; AmB, amphotericin B; AmB-DOC, amphotericin B deoxycholate; ApoA-I, apolipprotein A-I; ASSP, acetylated *Sterculia striata* polysaccharide; AUC, area under the concentration-time curve; BBB, blood–brain barrier; BEC_50_, minimum biofilm eradication concentration; BS, bile salt; BUN, blood urea nitrogen; CAmB, cochleated amphotericin B; CC_50_, 50% cytotoxic concentration; CDI, carbodiimide; CH, chitosan; CMC, critical micelle concentration; *C*_max_, peak plasma concentration; CS, oligochitosan; DCP, dicetyl phosphate; DDS, drug delivery system; DMPC, dimyristoyl phosphatidylcholine; DMPG, dimyristoyl phosphatidylglycerol; DMSO, dimethylsulfoxide; DNA, deoxyribonucleic acid; DOPE, dioleoyl phosphatidylethanolamine; DOTAP, dioleoyl-3-trimethylammonium propane; DS, degree of substitution; DSHemsPC, distigmasterylhemisuccinyl phosphatidylcholine; DSPE, distearoyl phosphatidylethanolamine; EE, encapsulation efficiency; FDA, Food and Drug Administration; FPF, fine particle fraction; GCPQ, *N*-palmitoyl-*N*-methyl-*N*,*N*-dimethyl-*N*,*N*,*N*-trimethyl-6-*O*-glycol chitosan; GDL, glyceride dilaurate; GIT, gastrointestinal tract; GMO, glyceryl monooleate; GMS, glyceryl monostearate; GSD, geometric standard deviation; HI, hydrophobicity index; HPMC, hydroxypropylmethylcellulose; HSNE, human septonasal epithelial cells; IC_50_, half-maximal inhibitory concentration; IFNγ, interferon γ; IL, interleukin; i.v., intravenous; LAA, lipoamino acid; LAB, Labrasol^®^; LC_50_, 50% lethal concentration; LDH, lactate dehydrogenase; LNA, linolenic acid; LPNP, lipid–polymer nanoparticle; LPS, lipopolysaccharide; LYO, lyophilized; MCT, medium chain triglycerides; MFC, minimum fungicidal concentration; MIC, minimum inhibitory concentration; MLP, magnetic liposomes; MMAD, mass median aerodynamic diameter; MPEG, methoxy-poly(ethylene glycol); MRI, magnetic resonance imaging; MTT, 3-(4,5-dimethylthiazol-2-yl)-2,5-diphenyltetrazolium bromide; N/A, not available; NaC, sodium cholate; NaDC, sodium deoxycholate; NC, nanocapsule; ND, nanodisk; NE, nanoemulsion; NLC, nanostructured lipid carrier; OA, oleic acid; O/W, water-in-oil; PBS, phosphate buffered saline; PC, phosphatidylcholine; PEG, poly(ethylene glycol); PG, propylene glycol; PHY, phytantriol; PI, polydispersion index; PII, primary irritation index; PK, pharmacokinetic; PLGA, poly(D,L-lactide-co-glycolic acid); PS, phosphatidylserine; RBC, red blood cell; SDCS, sodium deoxycholate sulfate; SEDDS, self-emulsifying drug delivery system; SGF, simulated gastric fluid; SIF, simulated intestinal fluid; SL, sophorolipid; SLN, solid lipid nanoparticle; S_mix_, surfactant/co-surfactant ratio; SML, surface-modified liposomes; SNEDDS, self-nanoemulsifying drug delivery system; SPC, soy phosphatidylcholine; Sta, stearylamine; TEER, transepithelial electrical resistance; TEWL, transepidermal water loss; TGFβ, transforming growth factor β; TNF-α, tumor necrosis factor α; TPGS, D-α-tocopheryl polyethylene glycol succinate; UDL, ultradeformable liposomes; UV, ultraviolet; VL, visceral leishmaniasis; ZOI, zone of inhibition; ζ, zeta potential.

**Table 3:** Recently completed, on-going and expected clinical trials evaluating amphotericin B (AmB) safety and efficacy against antifungal and antiparasitic diseases.

Formulation	Phase	Trial Identifier (Acronym)	Population (*n*)	Treatment Regimen	Status, (Expected) Start Date–(Estimated) Completion Date
Topical AmB solution (2 mg/mL in 30% DMSO)	4	NCT03814343	Non-dermatophytes onychomycosis (*n* = 20)	1–3 drops of AmB solution (2 mg/mL in 30% DMSO) once daily vs. placebo (30% DMSO solution) for 12 weeks	Recruiting, January 2019–June 2020
Topical 3% AmB cream (Anfoleish^®^)	1b/2	NCT01845727	Uncomplicated CL in Colombia (*n* = 80)	Topical 3% AmB cream twice or thrice daily for 4 weeks	Completed, February 2014–July 2016
Topical 0.4% L-AmB gel	2	NCT02656797	CL due to *L. major* or *L. tropica* (*n* = 108)	Topical AmB 0.4% liposomal gel vs. placebo gel	Recruiting, January 2018–January 2021
L-AmB (AmBisome^®^) aerosol plus oral itraconazole	3	NCT03656081	Chronic pulmonary aspergillosis (*n* = 224)	Oral itraconazole (200 mg tablet twice daily) and inhaled L-AmB (25 mg) or inhaled placebo (isotonic saline) twice a week, for 24 weeks	Recruiting, December 2018–July 2023
Nebulized L-AmB (AmBisome^®^)	2	NCT02273661 (NEBULAMB)	Allergic broncho pulmonary aspergillosis, excluding cystic fibrosis (*n* = 174)	Aerosol of L-AmB at 25 mg once a week for 6 months vs. placebo (isotonic saline)	Active (not recruiting), November 2014–July 2019
L-AmB	4	NCT02686853	Cryptococcal meningitis without AIDS (*n* = 40)	Intrathecal administration of L-AmB	Unknown, January 2016–January 2018
L-AmB(AmBisome^®^) plus fluconazole	2/3	NCT03945448 (ACACIA)	Asymptomatic cryptococcal antigenemia in Uganda (*n* = 600)	AmBisome^®^ single i.v. dose (10 mg/kg) and/or fluconazole 800 mg for 2 weeks, 400 mg for 8 weeks and 200 mg up to 6 months	Not yet recruiting, May 2019–November 2023
L-AmB and follow-up oral itraconazole	2	NCT04059770	Disseminated histoplasmosis in AIDS patients (*n* = 99)	Induction therapy with L-AmB i.v. as single dose (10 mg/kg), two doses (10 mg/kg on day 1 and 5 mg/kg on day 3) or 3 mg/kg for 2 weeks, followed by oral itraconazole capsules (400 mg daily) for 1 year	Not yet recruiting, November 2019–October 2021
L-AmB (AmBisome^®^) monotherapy or plus miltefosine	3	NCT02011958	VL in HIV patients in Ethiopia (*n* = 59)	L-AmB i.v. (5 mg/kg/day on day 1, 3, 5, 7, 9 and 11) combined with oral miltefosine (one or two 50 mg capsules daily for 28 days) or alone (5 mg/kg/day on days 1 to 5, 10, 17 and 24)	Completed, July 2014–September 2017
L-AmB for injection 50 mg/vial (generic and AmBisome^®^)	1	NCT03636659	Bioequivalence study in VL patients under fed condition (*n* = 140)	L-AmB for injection (generic or AmBisome^®^) 50 mg/vial at 3 mg/kg/day i.v. infusion once daily for 5 days	Completed, May 2018–April 2019
L-AmB (AmBisome^®^)	4	NCT03311607	PKDL in Bangladesh (*n* = 280)	AmBisome^®^ 15 mg/kg over 15 days in 5 biweekly 3 mg/kg infusions	Completed, April 2014–October 2015
L-AmB (AmBisome^®^) plus oral miltefosine	2	NCT03399955	PKDL in Sudan (*n* = 110)	AmBisome^®^ 5 mg/kg/day i.v. infusion at day 1, 3, 5 and 7 combined with oral miltefosine twice daily (allometric dosing) for 28 days	Recruiting, May 2018–May 2022
AmB-DOC (Fungizone^®^) and follow-up voriconazole	4	NCT02283905 (BLASTO)	Pulmonary blastomycosis requiring mechanical ventilation (*n* = 6)	Continuously infused AmB-DOC (1 mg/kg/day) up to a total dose of 1 g, then stepped down to oral or i.v. voriconazole	Recruiting, June 2015–December 2020
AmB-DOC plus flucytosine	3	NCT04140461	HIV-associated cryptococcal meningitis (*n* = 40)	AmB (0.5 or 0.7 mg/kg IVGTT once daily) plus flucytosine (100 mg/kg orally once daily for 4 or 2 weeks)	Not yet recruiting, January 2020–April 2022
AmB-DOC plus flucytosine	1	NCT04072640 (TITOC)	Cryptococcal meningitis in HIV patients (*n* = 120)	Induction therapy with AmB-DOC (0.4–0.5 or 0.7–1.0 mg/kg/day i.v.) plus flucytosine (100 mg/kg/day) for 28 or 14 days, then fluconazole for consolidation (400 mg/day for 2 months) and maintenance (200 mg/day)	Not yet recruiting, January 2020–December 2022
Oral cochleate AmB (CAmB/MAT2203)	2	NCT04031833 (EnACT)	Cryptococcal meningitis in HIV patients in Uganda (*n* = 176)	Oral CAmB vs. standard i.v. AmB	Recruiting, October 2019–December 2021
Oral cochleate AmB (CAmB/MAT2203)	2a	NCT02629419 (CAmB)	Mucocutaneous candidiasis refractory or intolerant to standard non-intravenous therapies (*n* = 16)	Oral CAmB (200 mg, 400 mg or 800 mg)	Active (not recruiting), September 2016–December 2021
Oral cochleate AmB (CAmB/MAT2203)	2	NCT02971007	Vulvovaginal candidiasis (*n* = 137)	Oral CAmB (200 mg or 400 mg for 5 days) vs. oral fluconazole (150 mg single dose)	Completed, November 2016–May 2017

AIDS, acquired immunodeficiency syndrome; AmB, amphotericin B; AmB-DOC, amphotericin B deoxycholate; CAmB, cochelate amphotericin B; CL, cutaneous leishmaniasis; DMSO, dimethylsulfoxide; HIV, human immunodeficiency virus; i.v., intravenous; IVGTT, intravenous glucose tolerance test; L-AmB, liposomal amphotericin B; PKDL, post-kala-azar dermal leishmaniasis; VL, visceral leishmaniasis (kala-azar).
